# Tox_(R)CNN: Deep learning-based nuclei profiling tool for drug toxicity screening

**DOI:** 10.1371/journal.pcbi.1006238

**Published:** 2018-11-30

**Authors:** Daniel Jimenez-Carretero, Vahid Abrishami, Laura Fernández-de-Manuel, Irene Palacios, Antonio Quílez-Álvarez, Alberto Díez-Sánchez, Miguel A. del Pozo, María C. Montoya

**Affiliations:** 1 Cellomics Unit, Cell & Developmental Biology Area, Centro Nacional de Investigaciones Cardiovasculares (CNIC), Madrid, Spain; 2 Mechanoadaptation and Caveolae biology, Cell & Developmental Biology Area, Centro Nacional de Investigaciones Cardiovasculares (CNIC), Madrid, Spain; University at Buffalo - The State University of New York, UNITED STATES

## Abstract

Toxicity is an important factor in failed drug development, and its efficient identification and prediction is a major challenge in drug discovery. We have explored the potential of microscopy images of fluorescently labeled nuclei for the prediction of toxicity based on nucleus pattern recognition. Deep learning algorithms obtain abstract representations of images through an automated process, allowing them to efficiently classify complex patterns, and have become the state-of-the art in machine learning for computer vision. Here, deep convolutional neural networks (CNN) were trained to predict toxicity from images of DAPI-stained cells pre-treated with a set of drugs with differing toxicity mechanisms. Different cropping strategies were used for training CNN models, the nuclei-cropping-based Tox_CNN model outperformed other models classifying cells according to health status. Tox_CNN allowed automated extraction of feature maps that clustered compounds according to mechanism of action. Moreover, fully automated region-based CNNs (RCNN) were implemented to detect and classify nuclei, providing per-cell toxicity prediction from raw screening images. We validated both Tox_(R)CNN models for detection of pre-lethal toxicity from nuclei images, which proved to be more sensitive and have broader specificity than established toxicity readouts. These models predicted toxicity of drugs with mechanisms of action other than those they had been trained for and were successfully transferred to other cell assays. The Tox_(R)CNN models thus provide robust, sensitive, and cost-effective tools for *in vitro* screening of drug-induced toxicity. These models can be adopted for compound prioritization in drug screening campaigns, and could thereby increase the efficiency of drug discovery.

## Author summary

Visualization of nuclei using different microscopic approaches has for decades allowed the identification of cells undergoing cell death, based on changes in morphology, nuclear density, etc. However, this human-based visual analysis has not been translated into quantitative tools able to objectively measure cytotoxicity in drug-exposed cells. We asked ourselves if it would be possible to train machines to detect cytotoxicity from microscopy images of fluorescently stained nuclei, without using specific toxicity labeling. Deep learning is the most powerful supervised machine learning methodology available, with exceptional abilities to solve computer vision tasks, and was thus selected for the development of a toxicity quantification tool. Two convolutional neural networks (CNN) were developed to classify cells based on health status: Tox_CNN, relying on prior cell segmentation and cropping of nuclei images, and Tox_RCNN which carries out fully-automated cell detection and classification. Both Tox_(R)CNN classification outputs provided sensitive screening readouts that detected pre-lethal toxicity and were validated for a broad array of toxicity pathways and cell assays. Tox_(R)CNN approaches excel in affordability and applicability to other in vitro toxicity readouts and constitute a robust screening tool for drug discovery.

## Introduction

Toxicity is a major cause of failure in drug development and causes costly withdrawals of drugs from the market. Drug development productivity would be greatly improved if cytotoxic compounds were identified during early *in vitro* screening [1–4]. Drug-induced cytotoxic effects lead to changes in cell and nuclear morphology which are characteristic of the specific cell-death pathway involved, the best characterized being apoptosis, necrosis, and autophagy [5–7]. The field has advanced with the establishment of high content screening (HCS) techniques and the emergence of toxicity reporters revealing specific biochemical pathways triggered during cell death programs or measuring metabolic cell function [8–10]. However, toxicity reporters are often limited to assess the specific biochemical pathways for which they were designed [11–13], and they are thus unlikely to capture the wide variety of toxic effects that can be triggered by different drugs in screening campaigns. Toxicity-screening approaches have combined multi-parametric image analysis of fluorescently labeled nuclei with the use of toxicity reporters in advanced machine learning pipelines [14–18]. However, toxicity reporters increase experimental complexity, thus reducing throughput and increasing screening costs. There is therefore an urgent need to develop broad specificity, cost-effective *in vitro* toxicity assays for incorporation in the primary screening phases of drug development. Cytotoxic effects have classically been visually identified from cell and nuclear morphology [5–7]. However, the complexity and variability of toxicity-associated morphological patterns has so far hindered their systematic and quantitative analysis and thus prevented their use as standalone toxicity screening endpoints. Although nuclear fluorescence staining forms the basis of most high content cell-based assays, its use is normally limited to image segmentation and nuclei counting to score cell-loss due to lethal toxicity [19,20], thus disregarding the wealth of information contained in images of fluorescently labeled nuclei. In an effort to exploit this information for the quantification of pre-lethal toxicity, we have explored state-of the-art machine learning tools for automated pattern recognition. The success of classical learning-based computer vision methods relies heavily on extraction and selection of a reasonable set of relevant features that are highly discriminative of the phenotypes being studied. Feature extraction and selection requires in-depth knowledge of the phenotype under study, which is hindered in the current application by the complexity and great variety of drug-induced toxicity-associated nuclei organization patterns. The most recent major advance in machine learning is deep convolutional neural networks (CNN) which, similar to a brain, have multiple layers of interconnected artificial neurons [21,22]. Through an automated process, deep neural networks learn abstract representations of raw images from pixel information as a progressive hierarchy of sub-images, from which they extract features that can be used to classify complex patterns in a supervised manner. CNNs can thus automate the critical steps of feature extraction and selection by learning to extract high-level features based on spatial relationships, which has enabled them to outperform other machine learning methods in computer vision tasks, as demonstrated for several challenging biomedical applications [23–28]. Thus, deep technology seemed well suited to the analysis and prediction of drug toxicity in images of fluorescently labeled nuclei.

Here, we present novel deep-learning approaches for in vitro cell-based toxicity assessment. The Tox_(R)CNN approaches proved to efficiently predict a broad spectrum of toxicity mechanisms from different drugs, nuclear stains and cell lines. The main strength of these tools is their unique ability to predict toxicity based exclusively on nuclei staining; this offers the advantage of improved affordability and applicability of toxicity prediction. The attraction of the Tox_(R)CNN tools relies on their high potential to enable sensitive and efficient compound prioritization based on detection of pre-lethal toxicity in primary screening campaigns.

## Results

### CNN can predict cell toxicity based on images of fluorescently stained nuclei

**Fig 1:**
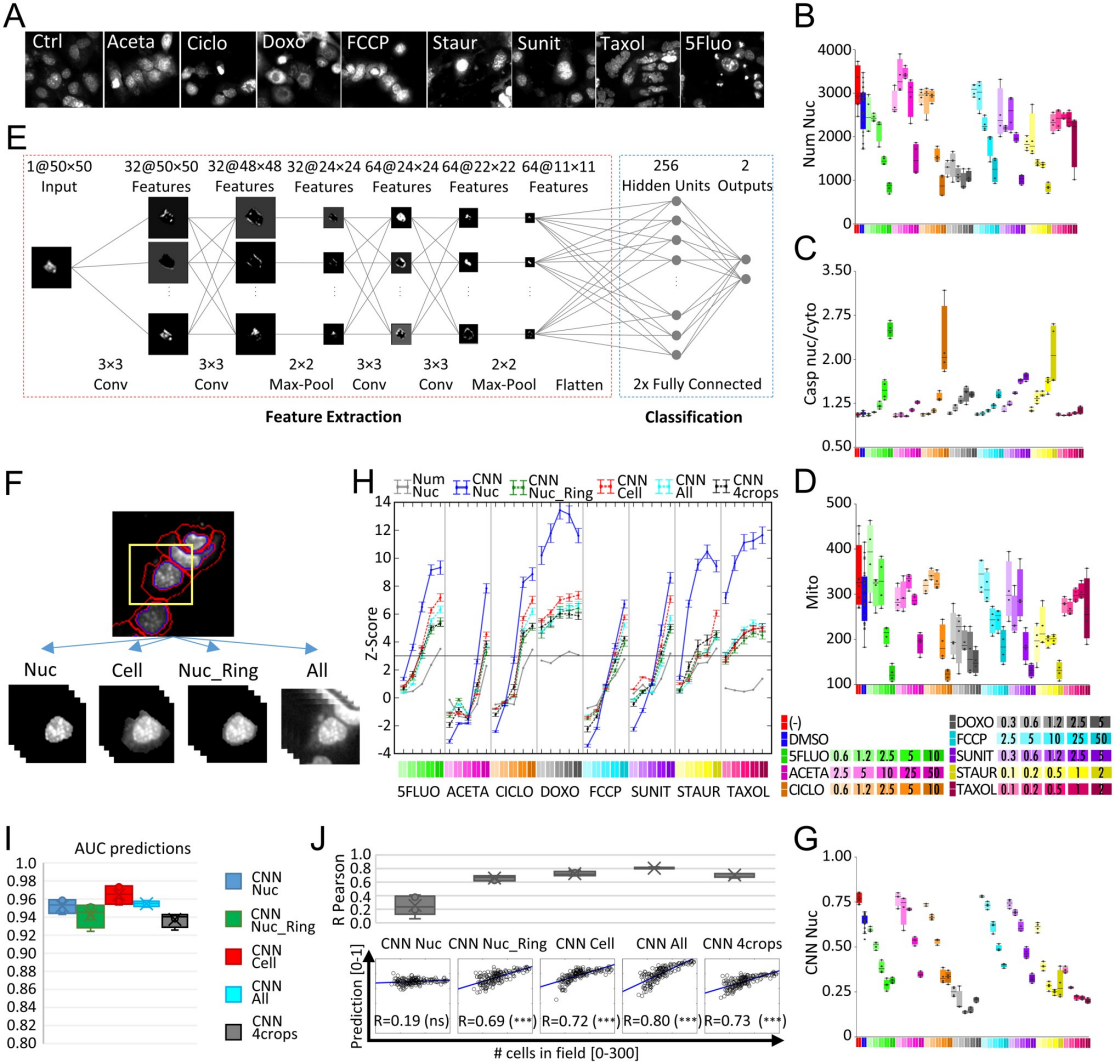
Toxicity prediction using deep CNN strategies compared with established readouts. HL1 cells treated or not (-) with the indicated concentrations of compounds (μM) or DMSO were processed as described in the Materials and Methods. (A) Representative fluorescence microscopy images of DAPI-stained cells treated or not (Ctrl) with the highest concentrations of the indicated compounds in a reference experiment (Experiment #1) used for CNN training. (B-D) Boxplots of per-well toxicity assessments from established measurements: nucleus count (Num Nuc) (B), Caspase 3/7 nucleus:cytoplasm ratio (Casp nuc/cyto) (C), and Mitotracker cytoplasmic intensity (Mito) (D). (E) CNN architecture for predicting health status from single-cell image crops, as described in Materials and Methods. (F) Cropping strategies; representative image crops are shown of nucleus (Nuc), nucleus+cytoplasm (Cell), nucleus+margin (Nuc_Ring), and nucleus+cytoplasm+background+neighboring cells (All). (G) Boxplot of per-well toxicity assessment from CNN Nuc predictions (percentage of cells classified as healthy). (H) Plot displaying mean toxicity readouts of replicate wells, obtained from the percentage of healthy cells predicted by the different CNN models (CNN Nuc, Nuc_Ring, Cell, All, 4crops) and the standard nuclei counting (Num Nuc) for the different treatments indicated. For each well, toxicity readouts were obtained by computing Z-scores (normalizing to DMSO-treated wells) with adjustment of the sign to display toxic effects as positive values. Points and corresponding error bars represent the mean and standard error of the mean, respectively, of results obtained by evaluating the 5 different CNN models trained for each cropping strategy. (I) Evaluation performance of the different CNN models for predicting toxic effects of staurosporine assessed using Caspase 3/7 fluorescent reporter as reference, as described in the Materials and Methods. Boxplots display AUC values obtained with the 5 models trained for each cropping strategy. (J) Correlations between cell density and CNN predictions obtained with the different models for untreated cells. Boxplots represent Pearson correlation coefficient, R, obtained with all 5 models trained with each cropping strategy (top), and exemplary dotplots for each strategy (selecting the model with the median AUC among the five) including regression line, R value and significance (bottom). p-value: *<0.01, **<0.001, ***<0.0001.

To test if CNNs can predict cell toxicity based exclusively on nuclear staining, we designed an experimental assay in which HL1 cells were treated with reference compounds at different concentrations. The included reference compounds cover a range of cytotoxic effects: the DNA targeting genotoxic drugs cyclophosphamide (Ciclo), 5-fluorouracil (5Fluo), and doxorubicin (Doxo); the apoptosis-triggering drug staurosporine (Staur); the enzyme inhibitors acetaminophen (Aceta) and sunitinib (Sunit), which are not associated with a specific toxicity mechanism; the uncoupler of mitochondrial oxidative metabolism FCCP; and the microtubule stabilizer Taxol, which inhibits mitosis. To guarantee varying degrees of toxicity outcome we used previously empirically established dose-curves by selecting concentrations of drugs ranging between having no effect up to significantly reducing cell number due to moderate cell death. Cells were labeled with the DNA-specific fluorescent probe DAPI and imaged with an automated confocal microscope, revealing a great variety of nuclear patterns induced by the different compounds (Fig 1A). The standard toxicity readout of nucleus count (Num Nuc) revealed cell loss due to drug-induced lethal effects, but did not reveal the great variety of toxic effects associated with the reference compounds assayed (Fig 1B). As reference toxicity readouts, we analyzed Caspase 3/7 nuclear translocation and Mitotracker cytoplasmic intensity (Fig 1C and 1D), both of which evidenced dose-dependent toxic effects promoted by the compounds tested. To assess toxicity independently of cell density and to enable detection of pre-lethal toxicity, we implemented a deep CNN architecture for the estimation of cell health status from microscopy images of DAPI-stained nuclei (Fig 1E). Since we are aiming at a cell-based toxicity assessment, we used standard image analysis procedures to segment nuclei and cytoplasm according to the DAPI signal and used the segmentation to crop images (see Materials and methods). Different image cropping strategies were designed based on the regions of interest included in the resulting image crop; nuclei (Nuc), nuclei and cytoplasm (Cell), nuclei and 3 adjacent pixels (Nuc_Ring), and nuclei, cytoplasm, and background (CNN All) (Fig 1F). Cropped images, each one containing a mass-centered cell, were used to train independent CNN models for making toxicity prediction (CNN Nuc, CNN Cell, CNN Nuc_Ring and CNN All). An additional model was trained using the combination of images obtained using the different cropping strategies (CNN 4crops). We repeated the training of the models (CNN Nuc, Nuc_Ring, Cell, All, and 4crops) 5 times each, in order to increase confidence in model comparison results and evaluate reproducibility. CNN models deliver a “health status” score as output for each cell, which determines a binary classification: *healthy* or *toxicity affected*. As a supervised learning technique, the CNN required images labeled according to the expected output (*healthy* or *toxicity affected*) as ground-truth images for training. However, the reference standards analyzed for this purpose, Caspase 3/7 and Mitotracker, did not qualify as general toxicity labels because neither efficiently captured all the drug-induced cytotoxic effects included in our experimental assay: both yielded poor resolution of Taxol-induced cytotoxicity and of the kinetic effects produced by Doxo and Aceta. As an alternative strategy, we produced a training dataset by labeling image crops according to the treatment exposure; cells from untreated wells were labeled *healthy*, whereas those from wells treated with the highest drug concentrations were labeled *toxicity affected* (S1A Fig). The percentage of cells classified as *healthy* by the different CNN models served as the per-well measurement of “general” toxicity. In spite of the high degree of uncertainty introduced into the impure training set, the CNN *healthy* predictions efficiently revealed dose-response toxicity curves for all the drugs tested in the assay (Fig 1G and S1B–S1E Fig), providing better resolution than those obtained from Num Nuc or the Caspase or Mitotracker readouts (Fig 1B–1D). At high doses of several toxicants, CNN models trained with information from nuclei only (CNN Nuc) underwent an unexpected drop in toxicity prediction. This was not observed in CNN models trained with cytoplasmic information (CNN Cell/Nuc_Ring/All/4crops), which displayed a steady increase in toxicity prediction due to their enhanced ability to reveal DNA release into the cytoplasm during necrosis induced by high drug concentrations.

**Fig 2:**
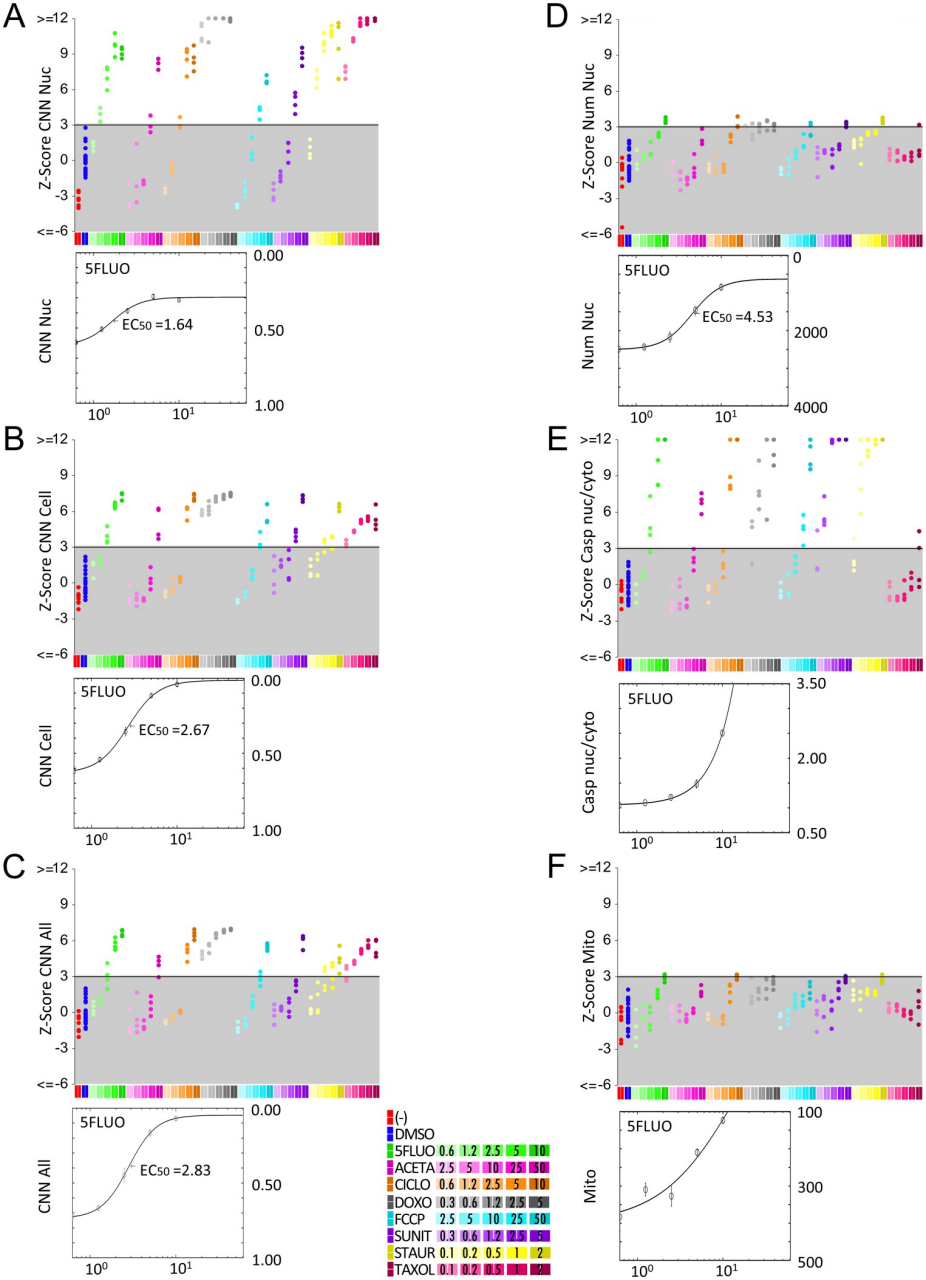
Sensitivity of CNN-based toxicity prediction compared with standard toxicity readouts. HL1 cells treated or not (-) with DMSO or the indicated concentrations of drugs (μM) from Experiment #1 were processed as described in the Materials and Methods. Plots display individual well toxicity readouts (top) and the 5-Fluorouracil dose-response curve fitted from well-averages, including the EC50 (bottom). Representative model results out of the 5 independent ones trained for each cropping strategy is shown. (A-C) CNN-based toxicity readouts: CNN Nuc (A), CNN Cell (B), and CNN All (C). (D-F) Standard toxicity readouts: Nuclei counting by standard image segmentation (Num Nuc) (D), mean Caspase 3/7 nucleus:cytoplasm ratio (Casp Nuc/Cyto) (E), and mean Mitotracker cytoplasmic intensity (Mito) (F). For each well, toxicity readouts were obtained by computing Z-scores (normalizing to DMSO-treated wells) with adjustment of the sign to display toxic effects as positive values. Z-scores > 3 represent toxic hits.

As screening readout, Z-scores were obtained from the percentage of cells classified as *healthy* by the CNN model for each compound-treated test well by normalizing to the DMSO-treated control cells (DMSO). Untreated cells were not used as negative controls since these are not commonly included as controls in screenings. To allow comparative evaluation of the different toxicity readouts and benchmarking the different cropping strategies, readouts are displayed so that Z-score positive values depict toxic effects. CNN readouts were more efficient at predicting toxicity than the classical Num Nuc readout (Fig 1H), with CNN Nuc displaying the highest Z-scores. Model performance was assessed on cells treated with the apoptosis-inducer staurosporine, which allowed the use of Caspase readout for ground truth labelling (i.e., Caspase-negative cells from untreated wells were labelled *healthy* whereas Caspase-positive cells from staurosporine-treated wells were labeled as *toxicity affected*). This test set was used for computing Receiver Operating Characteristic (ROC) curves to evaluate model prediction accuracies using the Area Under the ROC Curve (AUC) measurement. Results show high performance (above 0.9AUC) for all cropping strategies (Fig 1I), being the CNN Cell the most accurate. Importantly, CNN Nuc predictions were less correlated with the number of cells in the field than the other cropping strategies (Fig 1J), which displayed non-negligible correlations, suggesting that predictions of the health status of a single cell are influenced by surrounding information, including the presence of neighboring cells. As consequence, CNN Nuc reveals to be more independent of experimental errors due to cell density inconsistencies and also more indicated to detect pre-lethal toxicity. Accordingly, the CNN Nuc model proved to be the best readout for early prediction of toxicity, since it outperformed other cropping strategies at sub-lethal drug concentrations, where there is no reduction in Num Nuc indicating significant cell loss. Consistent with this pattern, plotting treatments by Z-scores to reveal above-threshold “toxic hits” confirmed CNN Nuc to be the most sensitive method for detecting early toxicity yielding 100 toxic hits (rounded mean of results from the 5 CNN Nuc models) out of 184 treated wells (Fig 2A), thus outperforming other CNN models. CNN Cell, Nuc_Ring, All and 4crops readouts yielded 82, 74, 77 and 83 hits, respectively (Fig 2B and 2C and S1A and S1B Fig). The standard toxicity readouts Num Nuc, Caspase and Mitotracker detected 27, 81, and 6 toxic hits, respectively (Fig 2D–2F). All CNN models yielded significant Z-scores for Taxol at 0.1*μ*M, demonstrating broader application than established readouts; Caspase detected toxicity effects only at 2*μ*M Taxol, whereas Mitotracker and Num Nuc did not detect significant Taxol toxicity. The higher sensitivity of CNN Nuc compared with the other CNN models and established readouts was further evidenced by the lower half-maximal toxic concentration values (EC50) obtained for the mild toxicant 5Fluo (Fig 2 and S2 Fig). Based on these results, nuclear crops were used as inputs for CNN models in all subsequent studies, and from here on CNN refers to these CNN Nuc models.

### Region-based CNN (RCNN) for fully automated toxicity prediction

**Fig 3:**
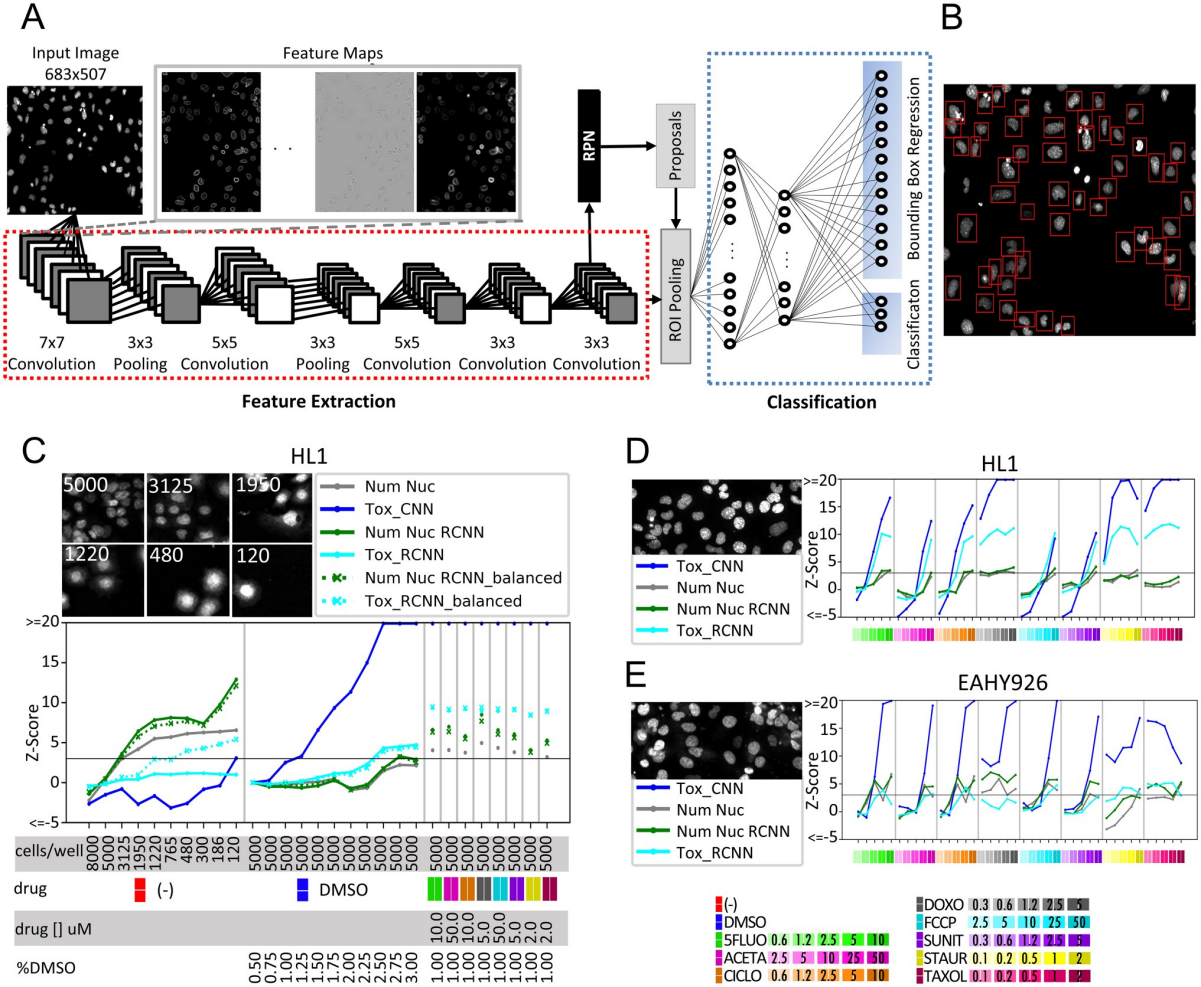
Region-based CNN implementation and evaluation of (R)CNN deep-learning toxicity-assessment approaches. (A) RCNN architecture for automated detection of cells and prediction of their health status from micrographs of DAPI fluorescence, as described in the Materials and Methods. (B) Example of nuclei bounding boxes resulting from the region proposal network included in the RCNN framework. (C) HL1 cells (Experiment #8) were seeded at the indicated densities (cells/well). (D,E) HL1 (Experiment #1) and EAHY926 (Experiment #9) cells were seeded at 5000 cells/well. (C-E) 24h after seeding, cells were treated or not (-) with the indicated concentrations of DMSO (%) or the indicated drugs (μM) and processed as described in the Materials and Methods. Representative images are shown of untreated cells at the indicated cell-seeding densities (cells/well). Plots display mean toxicity readouts of four replicate wells, obtained from the percentage of *healthy* cells predicted by the CNN Nuc (Tox_CNN) and RCNN (Tox_RCNN_balanced and Tox_RCNN) mixed models, and from nuclei counting by standard image segmentation (Num Nuc), or by using RCNN-based automated detection (Num Nuc RCNN) from Tox_RCNN training. For each well, toxicity readouts were obtained by computing Z-scores (normalizing to DMSO-treated wells) with adjustment of the sign to display toxic effects as positive values.

To avoid relying on external image segmentation and cropping procedures while providing a per-cell toxicity prediction, we undertook an alternative cell-based deep-learning approach incorporating automated object detection. An RCNN was implemented for the automated localization and classification of individual nuclei using raw images as input, instead of crops. The framework, based on the Faster RCNN algorithm [29], includes a region proposal network (RPN) that uses features extracted from the last convolutional layer of a CNN to detect bounding boxes around individual candidate cell, which are then classified as *healthy*, *toxicity-affected*, or *background* (Fig 3A and 3B). We trained the RCNN model with cell bounding box coordinates obtained with the standard segmentation procedure used in the CNN approach (see Materials and methods). A set of 7 independent experiments in HL1 cells treated with the eight reference drugs, including two experiments in which untreated cells were cultured at different confluencies, was used for in-parallel training of the RCNN and CNN mixed models for toxicity prediction using balanced datasets of *healthy* and *toxicity affected* labeled nuclei (Tox_CNN and Tox_RCNN_balanced). Resulting CNN predictions showed that the Tox_RCNN_balanced model, unlike Tox_CNN, erroneously predicted toxicity of untreated cells grown at low densities (Fig 3C). To prevent the models from learning to predict toxicity as a reduction in cell number due to drug-induced lethal effects, we trained an additional mixed model that included extra images of untreated cells cultured at different densities (120 extra *healthy* training wells), hereafter referred to as Tox_RCNN. The unbalanced and balanced Tox_RCNN models (Num Nuc RCNN and Num Nuc RCNN_balanced) detected a similar number of objects (nuclei); moreover, the number was consistent with the number obtained by the standard segmentation procedure (Num Nuc) at regular cell densities (Fig 3C–3E), demonstrating the efficiency of automated detection by RCNN. Tox_RCNN slightly overestimated cell number at low confluencies, probably due to the recognition of cellular debris that are discarded by the image processing procedure. Both Tox_CNN and Tox_RCNN mixed models successfully classified untreated cells at very low densities as *healthy* and efficiently predicted the toxic effects of drugs and high DMSO concentrations (Fig 3C), further demonstrating their independence from cell-density fluctuations. Tox_(R)CNN models performed efficiently in the test wells from an experiment used for training (Fig 3D) and in one independent experiment with HL1 cells at higher confluency (S3A Fig). Overall, Tox_(R)CNN models were consistently more sensitive than Num Nuc at predicting drug toxicity, with Tox_CNN outperforming Tox_RCNN classification in most cases. Even though these models were trained in HL1 cells, they suitably predicted toxicity from these drugs in two other cell lines, EAHY926 (Fig 3E) and MEVEC (S3B Fig), thus confirming the applicability of Tox_(R)CNN models for the prediction of toxicity in different cell types. In addition, these models successfully predicted toxicity of HL1 cells labelled with Hoechst 3342 (S4 Fig). Together, these results demonstrate the robustness of deep-learning-based toxicity prediction with regard to inter-experimental and intra-experimental variability, thus confirming Tox_(R)CNN as powerful screening tools.

### Validation of Tox_(R)CNN models for toxicity prediction of different drugs and mechanisms of action

**Fig 4:**
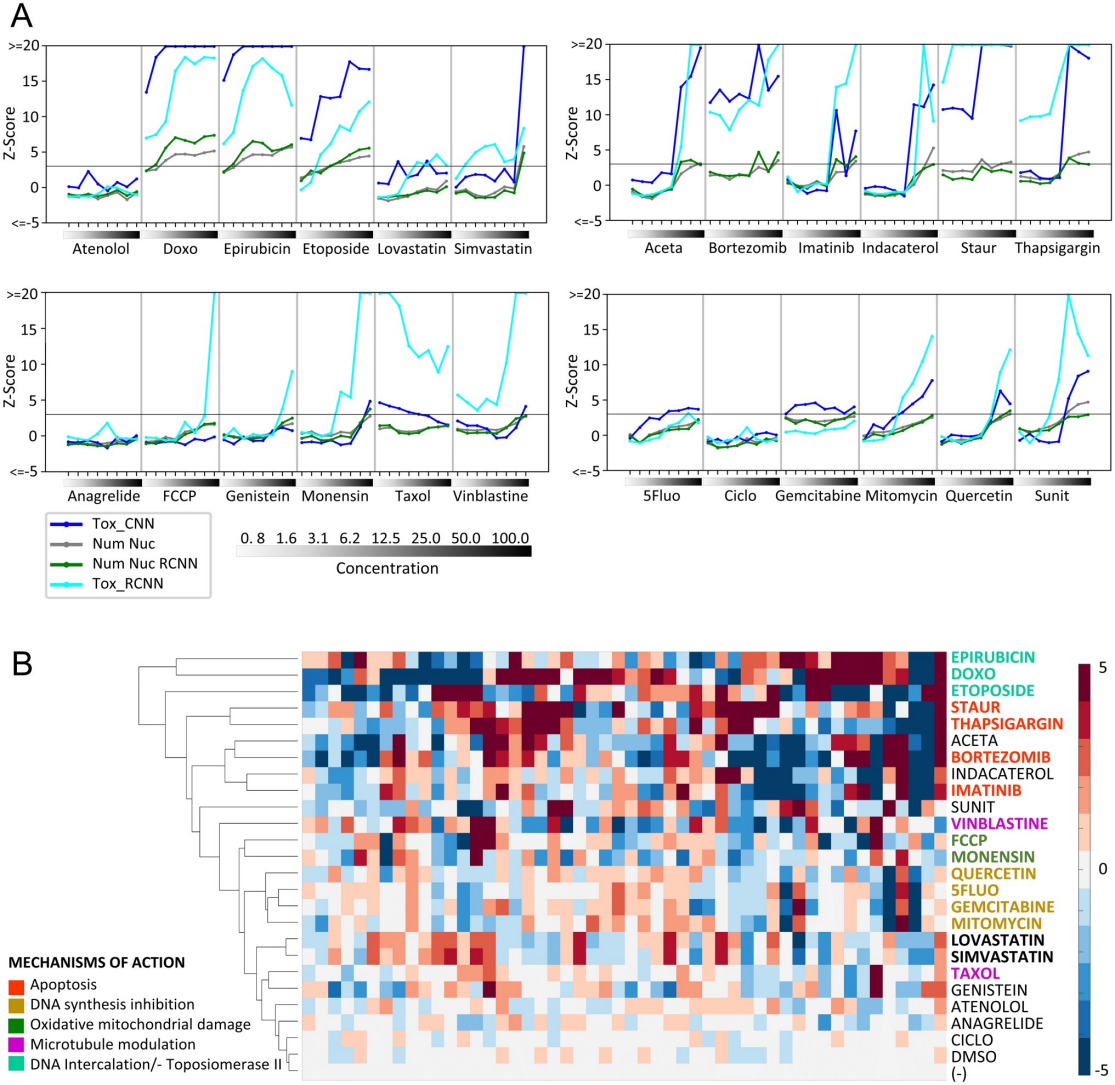
Deep (R)CNN model validation to predict toxicity and to extract knowledge for clustering of drugs based on their toxicity mechanisms. HL1 cells treated with one of 24 compounds or DMSO at the concentrations indicated (μM) (Experiments #11–14) were processed as described in the Materials and Methods. (A) Plots displaying mean toxicity readouts of four replicate wells, obtained from percentage of *healthy* cells predicted by the CNN Nuc (Tox_CNN) or RCNN (Tox_RCNN) mixed models, and from nuclei counting by standard image segmentation (Num Nuc), or by using RCNN-based automated detection (Num Nuc RCNN). For each well, toxicity readouts were obtained by computing Z-scores (normalizing to DMSO-treated wells) with adjustment of the sign to display toxic effects as positive values. (B) Hierarchical clustering of features obtained with the Tox_CNN model from HL1 cells treated with 25μM of the indicated drugs or 0.78μM Taxol; untreated (-); DMSO, control. Colors highlight mechanism of toxicity associated with compounds.

To demonstrate the value of these Tox_(R)CNN models as tools for broad toxicity prediction, we performed a new screening in which HL1 cells were treated with a panel of 24 drugs, including those used to train the CNN model and additional drugs acting through several mechanisms. Tox_(R)CNN mixed models sensitively predicted the outcome of toxic compound-treatments, thus proving their ability to reveal the toxicity of compounds for which they have not been trained (Fig 4A). Conveniently, Tox_CNN enabled the automated extraction of features from pixel intensity values, which were used for unsupervised hierarchical clustering of compounds (see Materials and methods). Tox_CNN features clustered compounds with known mechanisms of toxicity associated in a biologically meaningful manner, even though the models were not trained for this purpose (Fig 4B). The ionophores FCCP and monensin, which produce ROS and mitochondrial toxicity clustered together. The DNA synthesis inhibitors 5Fluo, gemcitabine, and mitomicine also group in the same cluster. Apoptotic death inducers (staurosporine, thapsigargin, bortezomib, and imatinib) were clustered closely together with other drugs of unknown mechanism. The DNA intercalating anthracyclines epirubicin and doxorubicin and the topoisomerase II inhibitor Etoposide, all of which promote double strand breaks also clustered together. Other drugs included, such as microtubule modulators Taxol and Vinblastine did not cluster together. Statins (lovastatin and simvastatin) with yet unknown mechanism of toxicity, but expected to be similar because belonging to the same family of proteins, were clustered together. These findings confirm not only that deep CNN are able to perceive general toxic effects, but also that their ability to learn feature representations provides useful knowledge for comparing drug-induced mechanisms of toxicity.

### Validation of Tox_(R)CNN models as toxicity screening tools

**Fig 5:**
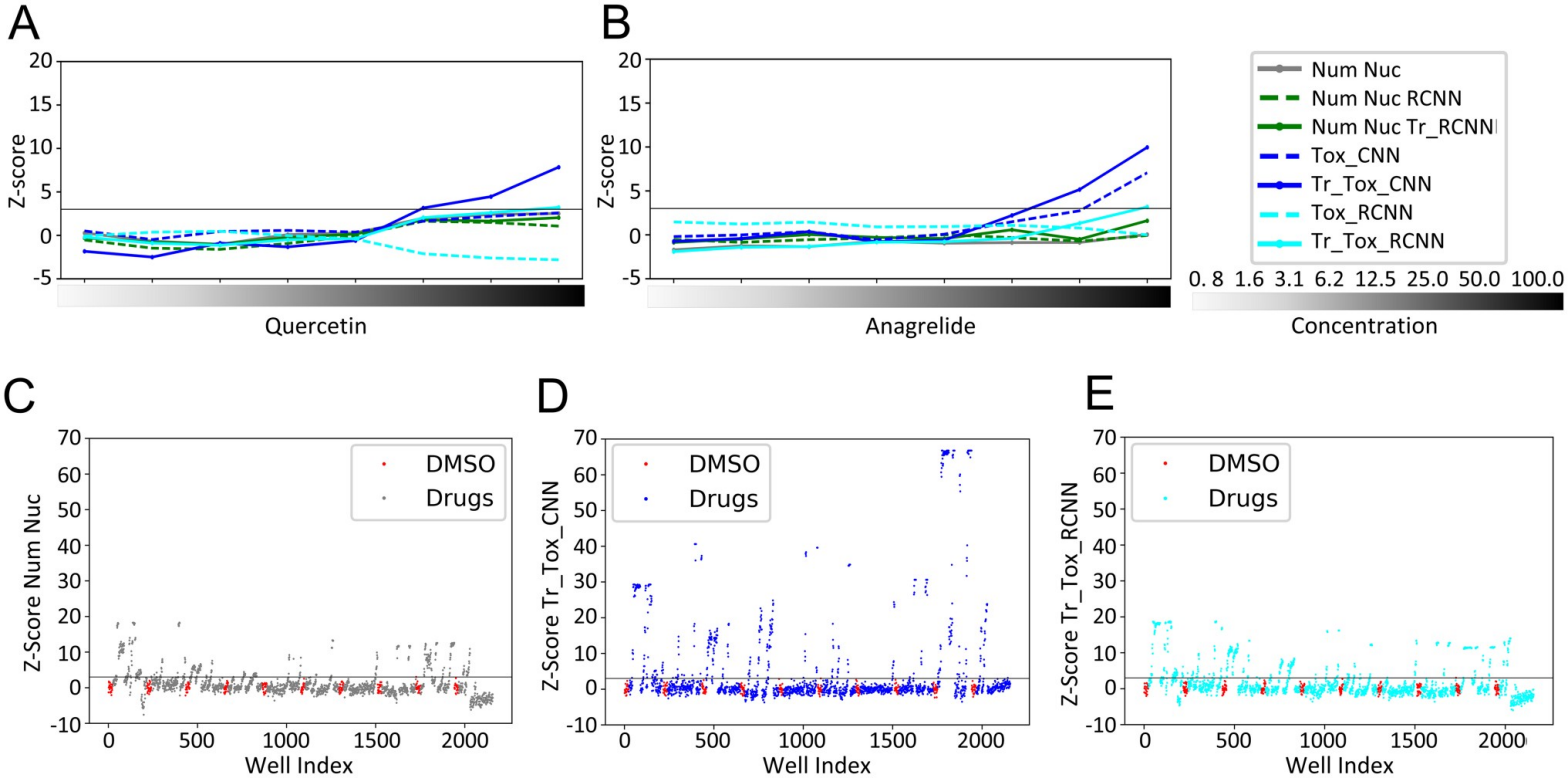
Validation of deep (R)CNN models for general drug toxicity screening. Pancreatic CAFs treated with 60 compounds or DMSO at the concentrations indicated (μM) (Experiments #15–24) were processed as described in the Materials and Methods. (A-B) Plots display mean toxicity readouts of replicate wells for quercetin (#19, A) and anagrelide (#33, B), obtained from the percentage of *healthy* cells predicted by the CNN Nuc (Tox_CNN) or RCNN (Tox_RCNN) mixed models before (dashed lines) and after (solid lines) transfer learning, and from nuclei counting by standard image segmentation (Num Nuc), or by using RCNN-based automated detection (Num Nuc RCNN) before (dashed lines) and after (solid lines) transfer learning. (C-E) Plots showing individual well toxicity readouts obtained after transfer learning from: nuclei counting using standard image segmentation (Num Nuc) (C), percentage of *healthy* cells predicted by CNN (Tr_Tox_CNN) (D), and percentage of *healthy* cells predicted by RCNN (Tr_Tox_RCNN) (E). Negative control, DMSO-treated wells, are highlighted in red. For each well, toxicity readouts were obtained by computing Z-scores (normalizing to DMSO-treated wells) with adjustment of the sign to display toxic effects as positive values.

To further evaluate the Tox_(R)CNN deep-learning models as screening tools for prioritizing compounds based on their toxicity potential, we re-analyzed a pre-accomplished HCS of primary pancreatic cancer associated fibroblasts (pan-CAFs). Among several assay-specific labels, most of which are irrelevant here, this HCS included DAPI staining in the assay for both image segmentation and nuclei counting as a toxicity endpoint. A transfer learning strategy was applied to the Tox_(R)CNN models delivering Tr_Tox_(R)CNN models. Training strategy was designed to allow the use of pre-run screens lacking reference toxicity-inducing treatments (see Materials and methods). In brief, the training dataset was produced from images from drug-treated wells with a significantly reduced cell number, which were labeled *toxicity affected*, while cells from DMSO treated wells were labeled *healthy*, since no untreated cells were available for training. Interestingly, compounds #19 and #33 (anagrelide and quercetin) were negligibly lethal according to nuclei counting, but were predicted by Tox_CNN to be toxic at high concentrations (Fig 5A and 5B), demonstrating the greater sensitivity of deep-learning-based prediction compared with Num Nuc. Toxicity of these compounds was confirmed by re-testing in primary cardiac fibroblasts (S5 Fig). Even though Tr_Tox_CNN performed better that the Tox_CNN model, the latter was still a more sensitive predictor of toxic effects than nuclei counting, demonstrating the value of these tools. Nuclei counting by both the transferred and original Tox_RCNN models (Tr_Tox_RCNN and Tox RCNN) was consistent with the standard procedure (Num Nuc), revealing that object detection was performing adequately. However, the original Tox_RCNN model was a poor toxicity predictor in pan-CAFs compared with the transferred RCNN model (Tr_Tox_RCNN), further evidencing the need for a transfer-learning approach for RCNN toxicity predictions in cell lines different from those used for training. The screening comprised 60 drugs at 8 concentrations (480 treatments) and yielded 102 toxicity hits (mean Z-score > 3) based on nuclei counting (Fig 5C). In contrast Tr_Tox_CNN and Tr_Tox_RCNN identified 127 and 126 toxic wells, respectively (Fig 5D and 5E). Z-scores computed for the transferred Tr_(R)CNN models were plotted independently for all compounds screened (S6 Fig). These results further demonstrate the superior performance and sensitivity of deep Tox_(R)CNN toxicity predictions over classical screening endpoints based on nuclei staining.

## Discussion

There is a need to incorporate highly predictive toxicity assays into primary in vitro high-throughput screening in order to reduce the attrition of drug candidates at later phases of the drug discovery pipeline. In vitro cytotoxicity assessment is normally limited to measuring the number of viable cells per well. However, single-cell readouts make better outputs that avoid sources of experimental error such as non-homogeneities in cell-dispensing, drug-induced proliferative effects, and heterogeneous responses of different cell sub-populations, which could be misinterpreted if only well averages are examined. The toxicity research field has therefore been directed towards finding novel cell labels and readouts that distinguish between different cytotoxicity mechanisms [8–13]. Nevertheless, the use of toxicity reporters has not gained broad acceptance because it adds experimental complexity, thus reducing throughput and increasing screening costs. Here, we have established tools that predict cell toxicity based on the analysis of fluorescently labeled nuclei. These tools outperformed outputs that rely on toxicity reporters or cell counting. Since nuclei staining is common to most high content cell based assays, this tool has broad applicability for toxicity prediction in HCS, even in pre-accomplished screens, as demonstrated here.

Over recent years, deep learning approaches have been successfully deployed in computer vision tasks and constitute the state-of-the-art tools for supervised machine learning. Several key features give deep learning approaches an advantage over other machine learning methodologies for toxicity analysis. First, by learning to represent data with multiple levels of abstraction in an unsupervised manner (i.e. without human-based programming) it avoids cumbersome knowledge-based feature engineering. This makes deep learning approaches independent of prior in depth knowledge of the target phenotypes, and therefore more suitable for broad toxicity prediction of multiple drugs with differing mechanisms by HCS. Second, their ability to learn intricate patterns improves recognition, feature extraction, and classification from noisy images, providing accuracy. This has led to deep technology outperforming other machine learning methods and human-based analysis, as demonstrated for several challenging biomedical applications [23–28]. Third, using transfer learning methods, networks can be updated to classify new datasets with limited training data [26,27,30], and thus makes them suitable for predicting toxicity in pre-accomplished screenings. Accordingly, the deep-learning approaches presented here successfully predict toxicity of a broad spectrum of toxicity-associated patterns in HCS images of fluorescently labeled nuclei, proving to be more sensitive than established toxicity reporters and readouts for the recognition of pre-lethal toxicity. The Tox_(R)CNN models suitably predicted toxicity in cell lines, nuclear stains and for compounds different from the ones used for training. Moreover, for very different cell lines, pre-trained Tox_(R)CNN models can be subject to a simple transfer-learning approach that does not require toxicity controls, increasing the performance of toxicity predictions; this was successfully achieved here in the pre-accomplished screening with primary pancreatic cancer associated fibroblasts (panCAF). Although CNN models were more sensitive and transferable and performed better, the use of RCNN models is justified by their independence from external segmentation and image cropping. We also demonstrate the utility of CNNs for extracting knowledge (as feature maps) that allows comparative analysis of different drugs in a screen. Previous cell-based toxicity screening approaches have combined multi-parametric image analysis of fluorescently labeled nuclei with the use of toxicity reporters in advanced machine learning pipelines [14–18]. The main strength of the tools presented here is their unique ability to predict toxicity based exclusively on nuclei staining, which offers the advantage of improving affordability and applicability of toxicity prediction. HCS is now an established tool for phenotypic drug discovery; in this setting, the deep-learning approaches presented here will promote a better use of HCS technology for toxicity assessment. The relevance of the deep learning approaches presented here relies on their high potential to enable sensitive and efficient compound prioritization based on detection of pre-lethal toxicity. They thus provide affordable cytotoxicity counter-screens for high throughput primary campaigns and could allow academic screening centers and pharma companies to discard cytotoxic compounds during primary screening and hit-to-lead drug development campaigns, thereby increasing the efficiency of drug discovery.

## Materials and methods

### Cell culture and reagents

Mouse cardiac muscle HL1 cells purchased from Merck Millipore were grown on fibronectin(25μg/ml)/gelatin(1mg/ml) coated dishes with 10% fetal bobine serum (FBS) in Claycomb medium (Sigma-Aldrich). Mouse embryonic ventricular endocardial cells (MEVEC) were kindly provided by Dr. de la Pompa [31] and cultured on 0.1% gelatin coated flasks in 10% FBS supplemented DMEM. EAHY926 cells were kindly provided by Dr. Edgell and maintained in 10% FBS supplemented DMEM. PanCAF were obtained from Dr. Hidalgo and cultured in RPMI with 20% FBS. Primary pig cardiac fibroblasts were isolated from fresh surgical samples by collagenase tissue digestion as described in [32]. Fibroblasts were grown to 80% confluency in flasks containing supplemented DMEM. All culture media were supplemented with 10% FBS (except PanCAFs, which had 20% FBS), 100 U/ml penicillin, 100 μg/ml streptomycin and 2 mM L-glutamine and refreshed every 2–3 days. Mycoplasma tests were performed bimonthly for all cell line cultures.

Fluorescence staining reagents including DAPI (4′, 6-diamidine-2-fenilindol), Hoechst 33342 (H42), Mitotracker Orange and Cell Event 3/7 Caspase Green, were purchased from Invitrogen. Dimethyl sulfoxide vehicle (DMSO); Acetaminophen (ACETA); Doxorubicin (DOXO); carbonylcyanide p-trifluoromethoxyphenylhydrazone (FCCP); Sunitinib; Staurosporine (STAUR); Paclitaxel (TAXOL); Imatinib; Thapsigargin; Gemcitabine; Quercetin; Atenolol; Simvastatin; Genistein; Vinblastine; Monensin; Anagrelide; Epirubicin; Etoposide and Lovastatin were from Sigma. Ciclophosphamide (CICLO), 5-Fluorouracil (5FLUO)and Sunitinib malate (SUNIT) were from Tocris. Indacaterol and Bortezomib were kinldy provided by Dr. Blanco, Experimental Therapeutics Programme at CNIO.

### Assay procedure

Cells were seeded on 384-well plates (5000 cells/well, otherwise specified), after 24h compounds were added to wells at N serial concentrations in 4 replicate wells. Plate was designed to avoid well distribution effects by using layout shown in S1A Fig, which places negative controls oriented asimetrically in distant positions within the plate (usually center and right). Additionally, in experiments designed for training deep learning models, double of wells with extreme doses were included in the plate simetrically distributed at top and bottom locations. To avoid evaporation-related edge effects external rows were filled with PBS and not used for the assay. Compounds were dissolved in DMSO (final concentration of 1% across the entire assay, otherwise specified). Cells were maintained in culture with compounds for 24h, and then stained for Caspase 3/7 and/or MitoTracker prior fixation with 4% PFA for 10 min at RT and final nuclei staining. Imaging of fluorescently labelled nuclei and toxicity probes was performed with an Opera automated confocal microscope (Perkin Elmer) fitted with an NA = 0.7 water immersion objective at a magnification of 20×. S1 Table summarizes all experiments used in this work, including information about cell lines, stainings, treatments, and number of images and cells.

### Image processing

Image processing algorithm was developed using Definiens Developer version XD2.4 (Definiens AG, Germany). Nuclei and cytoplasmic regions were first segmented based on differential contrast of DAPI/Hoechst 33342 intensity. Cells with a nuclear size bellow 0.3 times or over twice the mean of nuclear sizes per field were considered as debris and reclassified as non-cellular regions. An ID was univocally assigned to each cell the number of cells per well was computed. Toxicity readouts based on fluorescent reporters were extracted as Caspase 3/7 nucleus:cytoplasm intensity ratio (Casp nuc/cyto), and Mitotracker mean intensity in the cytoplasmic region (Mito), where specified.

Images from DAPI/Hoechst 33342 stained nuclei were cropped and saved individually assuring one cropped image per single mass-centered cell, thus conserving univocal cell ID. Four different strategies of cropping images were established (Fig 1F):

1. Nucleus (Nuc): Pixels from nuclear region preserve their intensity, while pixels from other regions including cytoplasm, neighbour cells or background are set to zero. Unless specified, this cropping strategy is used for training Tox_CNN models and classification purposes throughout the manuscript.
2. Nucleus and ring (Nuc_Ring): Pixels from nuclear region including a thin margin of 3 pixels containing a small region of the cell cytoplasm adjacent to the nuclei preserve their intensity. Pixels from other regions including the remaining cytoplasm, neighbour cells or background are set to zero. This cropping strategy was designed to include properties of nuclear border that could benefit the classification task.
3. Cell (Cell): Pixels from nuclear and cytoplasmic regions preserve their intensity. Pixels from other regions, including neighbour cells or background are set to zero. This cropping strategy incorporates information of nuclei fluorescence signal beyond the nuclear boundaries.
4. All regions (All): All pixels within the crop preserve their intensity, which include those from the cell nucleus and cytoplasm, the background and eventually the fractions of neighboring cells.

The four strategies were set to a fixed size of 50x50 pixel crops, thus guaranteeing a proper inclusion of complete nuclei based on nuclear sizes and image pixel sizes. To avoid off-centered nuclei crops, those nuclei with a distance to field image boundaries of less than 50 pixels were excluded from the cropping extraction and further analysis. Additionally, bounding box coordinates from segmented cells were also extracted for training automatic object detection by RCNN.

### Convolutional neural network (Tox_CNN)

We designed the toxicity convolutional neural network with an architecture and parameters adapted from a state-of-the-art CNN model, VGG [33], with high performance in image classification and well-known layers, activations, and initializations, including several 3x3 convolutions before a max-pooling layer. However, the limited size of our image crops (50x50 instead of 224x224 used by VGG) required a reduction in the depth of the network to prevent overfitting. Inspired by state-of-the-art architecture LeNet-5 [34], which was designed to work with 32x32 images, we constrained the network to 2 convolutional + max pooling groups of layers. Most of parameters were inherited from VGG model (batch size, initial learning rate, weight initialization…), since the lack of a general reference standard did not allow us to perform systematic optimization of parameters. Number of epochs was set up to 120 since performance of the network reached stability (hold-out validation using 33% of training data during 300 epochs with batch size of 256) while maintaining a reasonable computing time and preventing from potential overfitting. Kernel sizes of 3 and 5 were also tested reaching very similar results (AUC values for caspase-based evaluation were 0.9513±0.0065 and 0.9509±0.0053 for CNN Nuc model with kernel sizes of 3 and 5, respectively; p-val = 0.9), so we fixed them to 3 such as in original VGG architecture, which also speeded up computations x1.63. Final Tox_CNN network architecture comprises 4 convolutional and 2 fully connected layers (Fig 1E) to classify single-cell images as *healthy* or *toxicity affected*. The Rectified Linear Unit (ReLU) activation function is applied between each layer except the output dense layer, which uses a softmax activation function to provide a separate probability for each of the classes. We used ReLu as activation function since Sigmoid and Tanh can result in the so-called vanishing gradient problem. Convolutional layers convolve a 3×3 kernel over some input to produce 32, 32, 64, and 64 feature maps, respectively. To reduce the number of features and the computational complexity of the network, we introduced two max-pooling layers with a window size of 2×2 after convolutional layers 2 and 4. Additionally, to avoid overfitting, we included two dropout blocks after convolutional layers 2 and 4 (probability of 25%), and another one next to the first fully connected layer (probability of 50%). Dropout deactivates some neurons randomly with a specified probability during the weight update cycle. The final max-pooling layer is then flattened and followed by two densely connected layers with 512 and 2 features. Finally, we applied a softmax activation function to the output of last fully connected layer to calculate the probability for each class label. The total number of parameters to learn is equal to 4,031,458, most of them belong to the first fully connected layer. We used ADADELTA algorithm [35] to adjust the learning rate automatically. To increase the number of data and avoid overfitting, we augmented images by applying random rotations in the range of [0°, 20°], horizontal shifts in the range of [0, 0.2 × *Image*_*width*_], vertical shifts in the range of [0, 0.2 × *Image*_*height*_], and horizontal/vertical flips, where *Image*_*width*_ and *Image*_*height*_ show width and height of input images, respectively. By default, the modifications were applied randomly, so not every image will be changed every time. Images were normalized to zero mean and unit variance before feeding them into the network.

### Region based convolutional neural networks (Tox_RCNN)

We used state-of-the-art Faster Region-Based CNN [29] (RCNN) to both detect and classify cells as *healthy* or *toxicity affected* from entire images. Faster RCNN is composed of two modules: a Regional Proposal Network (RPN) and a RCNN network. RPN is a Fully Convolutional Network (FCN) [36] which proposes square regions within an image that may contain objects of interest without considering their classes while RCNN network classifies the object proposals from RPN into one of the classes (or background), and refines the bounding boxes’ coordinates of the final proposals. We used the original Faster RCNN architecture [29] without any significant modifications except for the number of outputs for the classification and regression layers since we have two classes. Therefore, the classification layer has 18 (9×2) outputs, and the regression layer has 36 (9×4) outputs (coordinates). Number of iterations was fixed to 750,000.

### Label-free toxicity annotation of images

To train the network for single-cell toxicity prediction, a set of cells need to be labelled as *healthy* and *toxicity affected*. The uncertainty of the toxicity state of individual cells due to lack of bona-fide toxicity reporters hamper the possibility of creating a pure and clean training dataset. Therefore, cells were labeled according to the known or expected toxic response of different extreme treatments; a set of untreated cells (or cells under harmless treatment) were labelled as *healthy*, and a set of cells treated with known toxic compounds at the highest concentrations were labelled as *toxicity affected*. This labelling strategy minimize the amount of manual supervision needed to perform the tedious task of creating a large annotated dataset for training, avoiding also the use of any toxicity labels such as fluorescent reporters that always provide partial information since they are unable to detect all the toxic effects. These *healthy* and *toxicity affected* sets are initially conformed by all cells in a selection of wells correspondent to the appropriate extreme treatments. Training sets for both classes (*healthy* and *toxicity affected)* were balanced, where indicated, by removing all cells from randomly selected entire images. The final output is a field-based treatment-driven training dataset that represents two groups/classes with the (expected) highest mean difference in terms of toxicity state. A training-strategy avoiding overfitting was undertaken to properly deal with minimized, yet still present mislabeling of cells affected by toxicity in conditions with harmless or no treatment, as well as resilient cells in extreme harmful conditions.

### Training Tox_(R)CNN

Training of both Tox_CNN and Tox_RCNN models need the outputs obtained from the image processing routine developed in Definiens (see Image processing section). CNN approach used cropped single cell 50 × 50 pixel images; and RCNN approach used full fields (683 x 507 pixel images) and bounding-box coordinates of cells. The initial experiment pursuing the comparison of CNN performance from the different cropping strategies used images from one experimental plate (Experiment #1), where the correspondent trainings were performed in parallel and repeated five times. The creation of central Tox_(R)CNN mixed models involved images from 7 HL1 experiments (Experiments #1–7) including several conditions: untreated cells, cells treated with DMSO, and cells treated with up to 8 drugs with known toxic effects at different concentrations. All drugs used demonstrated toxic effects in previous experiments where dose curves were fixed. Three of these experiments were designed in a way that allows the classifier to learn that healthy cells can also grow at low densities. Training dataset was created as detailed in the previous section, labelling single cells from wells without any treatment as *healthy*, and cells from treated wells with highest concentrations of the 8 available compounds as *toxicity affected*. In each plate, only half of these wells per condition were sparsely selected for training, and the rest were bound to test (S1A Fig). In total, Tox_CNN mixed model was trained using 739,727 cells (image crops) covering 8 compounds (*toxicity affected*) and untreated cells (*healthy*). Bounding-box coordinates of training cells, together with the correspondent 7,489 (balanced) and 10,883 (unbalanced) entire images (fields) were used to train the Tox_RCNN mixed models. S2 Table summarizes the number of instances (crops or field images) and experiments used for training each model.

### Transfer learning Tr_Tox_(R)CNN

For CAF screening, we used deep transfer learning [37] to adapt Tox_(R)CNN mixed models to a different cell line improving toxicity prediction. With this strategy, an existing pre-trained network is fine-tuned, avoiding training an entire neural network from scratch and reusing low level feature-detectors already learned. Therefore, we froze the weights of the first two convolutional layers in both (R)CNN approaches and retrained the rest of the layers to adapt to this new dataset. For this screening, since there is no prior information about the dose-response curve and expected toxicity, we used a different strategy to create the training set, but again following the guidelines detailed before (see Label-free toxicity annotation of images). First, we selected drugs with at least one concentration with a significant toxic effect scored as a significant reduction of the number of cells per well (Z-score>3). Then, for each selected drug, cells treated with the two highest concentrations of that drug were included in the training set, and labelled as *toxicity affected*. Since untreated wells are not included in regular screenings, cells from half of the DMSO-treated wells were included in the *healthy* training set, which corresponds to the harmless condition in this specific screening. This resulted in a training set with 150,529 instances (6,057 field-images). S2 Table includes information about the number of instances (crops or field images) and experiments used to create the transferred models. We followed this general strategy for generating training sets, to ensure that it can be used in any assay where no prior information about toxic effects of compounds included in the screening is available. We used 25 epochs to re-train the Tox_CNN model, and 45,000 iterations to update the weights of the Tox_RCNN model; conforming the transferred models for toxicity prediction in PanCAF screening (Tr_Tox_(R)CNN models).

### Tox_(R)CNN classification and evaluation

We evaluated the performance of Tox_(R)CNN models on several independent experiments. Cells out of the training set coming from experiments that were partially used for training were also employed for testing purposes: treatments with intermediate concentration of drugs were never included in training set, and not all wells from extreme treatments were selected for training. Tox_CNN model classified crop images at the input as *healthy* or *toxicity affected* based on the probabilistic scores obtained at the output for both classes. Tox_RCNN model return object detections which were classified as *healthy*, *toxicity affected* or *background* (considered as non-cell detections and discarded from further analyses) in entire field images. S3 Table summarizes experiments and number of instances (crops or field images) tested with the different models, and references to figures including the corresponding results.

### Toxicity readouts

Well-based toxicity measurements were constructed from Tox_(R)CNN predictions by computing the percentage of cells classified as *healthy* in each well. Standard toxicity measurements obtained from fluorescent reporters (Caspase 3/7 and Mitotracker) were aggregated in well-based values by computing the mean. Nuclei count obtained by image processing and RCNN-derived counting of nuclear detections were also reported for each well. For each type of toxicity measure *(x)*, we computed Z-scores by subtracting the mean (*μ*) and then dividing by the standard deviation (*σ*) of negative control (DMSO treated wells):

$$Z-Score=\frac{x-\mu }{\sigma }$$

Finally, we adjust the sign of the outputs to get increasing values for toxic effects, thus obtaining final well-based toxicity readouts that allow direct comparisons.

### Comparison of CNN models

The previously explained uncertainty of the toxicity status of individual cells under the wide range of different compound treatments hampers the proper evaluation of CNN models for parameter optimization and comparison of models obtained with different cropping strategies (CNN Nuc, CNN Nuc_Ring, CNN Cell, CNN All, and the combination of all the previous, CNN 4crops). For that reason, assessment of model performance was tackled in different ways:

1. Reproducibility. In order to increase confidence on results and measure variabilities, 5 independent models for each of the different cropping strategies were created (rounds) from 5 different initial states. In each round, the different models corresponding to each cropping strategy or combination of them were trained in parallel from the same initial state.
2. Consistency. Z-score readouts provide a measurement of distance in terms of number of standard deviations from the mean effect in control conditions. These values represent a magnitude that allow us to check the strength of toxic effects and the consistency in the evolution of these effects with drug doses in a meaningful manner.
3. Sensitivity. The ability of predicting early toxicity can be quantified by computing toxic hits (effects over a specific magnitude). The choice of the cutoff of Z-score = 3 has been previously suggested for hit selection in high throughput screening reports [38], which highlights that a condition with a Z-score bigger than 3 guarantees the fulfillment of the 3-sigma Rule [39]. Therefore, a Z-score of 3 translates into the distance of 3 × std extended from sample mean (or median), and the wells with a Z-score of 3 or greater represent an extreme 0.27% sub-set in a normal distribution [40]. This cutoff is not perfect since it is not robust to outliers as it tends to miss weak hits in data with frequent outliers, whereas lowering the standard deviation threshold to capture weak hits may unacceptably increase the rate of false positives [40,41]. Nevertheless, this threshold is found in the literature as a common cutoff for high-throughput screens [42,43] who reported that setting this threshold of 3 yielded a 0.5% hit rate.
4. Accuracy. A caspase-based evaluation can be used to measure the correctness of prediction: when toxic effects on cells are mainly driven by apoptotic processes, the ability to predict single cell toxic effects can be assessed using Caspase 3/7 fluorescent reporter. Therefore, we used caspase readout for ground truth labelling of untreated or staurosporine (an apoptotic death inducer) treated cells. To do so, we established a threshold of caspase positive cells using the distribution of Caspase nuc/cito ratio average value from untreated cells, plus three standard deviations. This allowed us to use caspase readout for ground truth labelling (i.e., caspase-negative cells from untreated wells as healthy and caspase-positive cells from staurosporine treated wells as toxicity affected). The corresponding test set was used for computing ROC curves to evaluate model prediction accuracies using AUCs.
5. Robustness. Single cell predictions should be independent on cell density. Although measuring toxic effects of compounds has been traditionally bound to cell density and nuclear counting, the presented CNN models pursue the prediction of toxicity at individual cell level, using only morphological information provided by nuclear staining. Therefore, each cell prediction should be independent on its neighborhood, providing a robust estimation not biased by neighboring cells to avoid confusing cell density changes with toxic effects. We measured this independence by computing Pearson correlations between number of cells in each field image and their mean predictions. This measurement was carried out in images from untreated cells, since these cells should be predicted as *healthy* independently on the amount of cells detected in the image.

### Hierarchical clustering

Tox_CNN features from the first fully connected layer (512 features/sample) were extracted for all cells in 4-plate screening of HL-1 cells with 24 drugs. Features obtained from cells that were either untreated, DMSO-treated, or treated with a non-extreme toxic concentration of the drugs (25 μM) (except for Taxol in which a 0.781 μM concentration was used) were analyzed by PCA to select the 50 most informative features for further processing. Mean well-based features were later normalized (Z-score with respect to untreated condition) and aggregated in order to obtain mean values per treatment/condition. Finally, these feature vectors were assembled by hierarchical clustering [44] to generate the hierarchical tree (clustergram function in MATLAB with Euclidean distance metric and weighted average linkage). Clusters along both dimensions (features and treatment/condition) were displayed in a heatmap of feature vector values including dendrograms representing the multilevel hierarchy obtained.

### EC50

Half maximal effective concentration (EC50) is the concentration of a drug which induces a response halfway between the observed baseline and maximum effect of that drug. For the present work, the drug responses used for the EC50 calculations are the different toxicity effects, as indicated. EC50 were computed by fitting a Hill Equation sigmoid curve to the dose-response values and estimating the correspondent EC50 Hill Equation parameter [45]. Dose-response values and adjusted curves are displayed in Fig 2 and S2 Fig in such way that visual increasing values depict toxic effects for all toxicity measurements in order to allow proper comparison. Response axes are fixed to [1; 0] for CNN predictions, [4,000; 0] for nuclei count, [0.5; 3.5] for the Caspase ratio and [500; 100] for Mitotracker measurements. EC50 values were not calculated in cases where standard sigmoidal curve fitting was inappropriate within the existing range of drug doses (Fig 2E and 2F).

### Software and hardware resources

We used Keras [46] on the Theano backend [47] to develop the Tox_CNN model presented here. To construct the Tox_RCNN model we used a python implementation of Faster RCNN [29] which is developed on Caffe [48]. All experiments were run on a standard PC with an Intel Xeon CPU E5-2643 @ 3.30 GHz, 32 GB working memory, and using a 16 GB NVIDIA Quadro K4000 GPU to speed up the computations. Training time for the Tox_CNN model was 11 hours; transfer learning took 2 hours. Training time for the Tox_RCNN mixed model and transfer learning were 187 hours and 9 hours, respectively. Boxplots and well-based dot plots were created with NCSS Statistical Software (version 11), and mean Z-score plots were depicted with Python. MATLAB (R2017a) was used to perform hierarchical clustering and to compute dose-response curve adjustments and EC50 calculations.

### Figures

All figures displaying (R)CNN-derived information include only results from test sets; otherwise indicated. Figures showing information from initial CNN models associated with different cropping strategies show either the results from 5 independently trained models or the results of a representative model/cropping-strategy performing with the median AUC value (out of five).

## Supporting information

S1 Fig Plate layout and predictions with secondary CNN strategies. (A) Plate layout corresponding to a reference experiment used for both training and testing CNNs, where cells from indicated wells were used to create the training dataset with *healthy* (green) and *toxicity affected* (red) labelled cells coming from untreated wells and wells treated with the highest drug concentrations, respectively. (B-E) HL1 cells treated or not (-) with DMSO or the indicated concentrations of drugs (μM) from Experiment #1 were processed as described in the Materials and Methods. Boxplots of per-well toxicity assessments from CNN-based predictions: percentage of cells classified as *healthy* by the CNN Nuc_Ring (B), CNN Cell (C), CNN All (D), and CNN 4crops (E) models.
(TIF)

S2 Fig Sensitivity of CNN Nuc_Ring and 4crops toxicity predictions. HL1 cells treated or not (-) with DMSO or the indicated concentrations of drugs (μM) from Experiment #1 were processed as described in the Materials and Methods. Plots display individual well toxicity readouts (top) and the 5-Fluorouracil dose-response curve (bottom), including the EC50, from CNN Nuc_Ring (A) and CNN 4crops (B) toxicity predictions. For each well, toxicity readouts were obtained by computing Z-scores (normalizing to DMSO-treated wells) with adjustment of the sign to display toxic effects as positive values. Z-scores > 3 represent toxic hits.
(TIF)

S3 Fig Evaluation of (R)CNN deep-learning toxicity-assessment approaches. HL1 (A) and MEVEC (B) cells treated or not (-) with DMSO or the indicated concentrations of drugs (μM) were processed as described in the Materials and Methods (Experiments #2 and #10). Representative images are shown of untreated cells. Plots display mean toxicity readouts of four replicate wells, obtained from the percentage of *healthy* cells predicted by the CNN Nuc (Tox_CNN) or RCNN (Tox_RCNN) mixed models, and from nuclei counting by standard image segmentation (Num Nuc), or by RCNN-based automated detection (Num Nuc RCNN). For each well, toxicity readouts were obtained by computing Z-scores (normalizing to DMSO-treated wells) with adjustment of the sign to display toxic effects as positive values.
(TIF)

S4 Fig Evaluation of a different nuclear staining. HL1 cells treated or not (-) with DMSO or the indicated concentrations of drugs (μM) were stained in parallel with DAPI (Experiment #26) or H42 (Experiment #27) as described in the Materials and Methods. Representative images of untreated cells are shown. Plots display toxicity readouts of four replicate wells, obtained from the percentage of *healthy* cells predicted by the CNN Nuc (Tox_CNN) or RCNN (Tox_RCNN) mixed models for both experiments. For each well, toxicity readouts were obtained by computing Z-scores (normalizing to DMSO-treated wells) with adjustment of the sign to display toxic effects as positive values.
(TIF)

S5 Fig Confirmation of (R)CNN-predicted toxic hits. Primary cardiac fibroblasts (Experiment #25) treated or not (-) with DMSO or the indicated concentrations of drugs (μM) were processed as described in the Materials and Methods. Boxplots of per-well toxicity assessments in culture wells from established measurements (A-C), and corresponding individual well toxicity readouts (D-F), obtained from Caspase 3/7 nucleus:cytoplasm ratio (Casp Nuc/Cyto) (A,D), Mitotracker cytoplasmic intensity (Mito) (B,E), and nuclei counting (Num Nuc)(C,F). Data are from 4 replicate wells of the same experiment. For each well, toxicity readouts (D-F) were obtained by computing Z-scores (normalizing to DMSO-treated wells) with adjustment of the sign to display toxic effects as positive values.
(TIF)

S6 Fig Validation of (R)CNN as drug toxicity screening tools. Pancreatic CAFs (Experiments #15–24) treated with 60 compounds at the indicated concentrations (μM) were processed as described in the Materials and Methods. Plots correspond to results in all 10 complete plates, displaying mean toxicity readouts of four replicate wells, obtained from the percentage of *healthy* cells predicted by the CNN (Tr_Tox_CNN) and RCNN (Tr_Tox_RCNN) mixed models after transfer learning, and from nuclei counting by standard image segmentation (Num Nuc), or by RCNN-based automated detection (Num Nuc Tr_RCNN). For each well, toxicity readouts were obtained by computing Z-scores (normalizing to DMSO-treated wells) with adjustment of the sign to display toxic effects as positive values.
(TIF)

S1 Table Experiments. Summary of all experiments used in this work, including information about cell lines, treatments, and the number of images and cells.
(XLSX)

S2 Table (R)CNN models and training. Summary of the number of instances (crops or field images) and experiments used for training each model generated in this work.
(XLSX)

S3 Table (R)CNN tests and figures. Summary of experiments and the number of instances (crops or field images) tested with the different models, and references to figures including the corresponding results.
(XLSX)

S1 File Supporting data. Compressed file containing processed data represented in figure’s graphics.
(ZIP)

## References

[1] Paul SM, Mytelka DS, Dunwiddie CT, Persinger CC, Munos BH, Lindborg SR, et al How to improve RD productivity: The pharmaceutical industry’s grand challenge. Nat Rev Drug Discov. 2010;9(3):203–14. 10.1038/nrd3078 20168317 10.1038/nrd3078

[2] Sunita SJ, Huang R, Austin CP, Xia M. The Future of Toxicity Testing: A Focus on In Vitro Methods. Using a Quantitative High Throughput Screening Platform. Drug Discov Today. 2010;15:997–1007. 10.1016/j.drudis.2010.07.007 20708096 10.1016/j.drudis.2010.07.007 PMC2994991

[3] McKim JM. Building a tiered approach to in vitro predictive toxicity screening: a focus on assays with in vivo relevance. Comb Chem High Throughput Screen. 2010 2;13(2):188–206. 10.2174/138620710790596736 20053163 10.2174/138620710790596736 PMC2908937

[4] Slater K. Cytotoxicity tests for high-throughput drug discovery. Curr Opin Biotechnol. 2001;12(1):70–4. 11167076 10.1016/s0958-1669(00)00177-4

[5] Orrenius S, Nicotera P, Zhivotovsky B. Cell death mechanisms and their implications in toxicology. Toxicol Sci. 2011 1;119(1):3–19. 10.1093/toxsci/kfq268 20829425 10.1093/toxsci/kfq268

[6] Kerr JFR, Wyllie AH, Currie AR. Apoptosis: A basic biological phenomenon with wide-ranging implications in tissue kinetics. Br J Cancer. 1972;26:239–57. 4561027 10.1038/bjc.1972.33 PMC2008650

[7] Trump BE, Berezesky IK, Chang SH, Phelps PC. The Pathways of Cell Death: Oncosis, Apoptosis, and Necrosis. Toxicol Pathol. 1997 1;25(1):82–8. 10.1177/019262339702500116 9061857 10.1177/019262339702500116

[8] Trask OJ. High Content Screening Methods in Molecular Biology. 2nd ed Johnston PA, Trask OJ, editors. Springer New York; 2018. 10.1007/978-1-4939-7357-6_23 29256175

[9] Tolosa L, Gómez-Lechón MJ, Donato MT. High-content screening technology for studying drug-induced hepatotoxicity in cell models. Arch Toxicol. 2015;89(7):1007–22. 10.1007/s00204-015-1503-z 25787152 10.1007/s00204-015-1503-z

[10] McGivern J V., Ebert AD. Exploiting pluripotent stem cell technology for drug discovery, screening, safety, and toxicology assessments. Adv Drug Deliv Rev. 2014;69–70:170–8. 10.1016/j.addr.2013.11.012 24309014 10.1016/j.addr.2013.11.012

[11] O’Brien P, Haskins JR. In Vitro Cytotoxicity Assessment. Methods Mol Biol. 2007;356:415–25. 16988420 10.1385/1-59745-217-3:415

[12] Kepp O, Galluzzi L, Lipinski M, Yuan J, Kroemer G. Cell death assays for drug discovery. Nat Rev Drug Discov. 2011;10(3):221–37. 10.1038/nrd3373 21358741 10.1038/nrd3373

[13] Pradip A, Steel D, Jacobsson S, Holmgren G, Ingelman-sundberg M, Björquist P, et al High content analysis of human pluripotent stem cell derived hepatocytes reveals drug-induced steatosis and phospholipidosis. Stem Cells Int. 2016;2016(2475631):1–14. 10.1155/2016/2475631 PMC4736406 26880940

[14] Abraham VC, Towne DL, Waring JF, Warrior U, Burns DJ. Application of a high-content multiparameter cytotoxicity assay to prioritize compounds based on toxicity potential in humans. J Biomol Screen. 2008;13(6):527–37. 10.1177/1087057108318428 18566484 10.1177/1087057108318428

[15] Pointon A, Abi-gerges N, Cross MJ, Sidaway JE. Phenotypic profiling of structural cardiotoxins in vitro reveals dependency on multiple mechanisms of toxicity. Toxicol Sci. 2013;132(2):317–26. 10.1093/toxsci/kft005 23315586 10.1093/toxsci/kft005

[16] Towne DL, Nicholl EE, Comess KM, Galasinski SC, Hajduk PJ, Abraham VC. Development of a high-content screening assay panel to accelerate mechanism of action studies for oncology research. J Biomol Screen. 2012 9;17(8):1005–17. 10.1177/1087057112450050 22706350 10.1177/1087057112450050

[17] Martin HL, Adams M, Higgins J, Bond J, Morrison EE, Bell SM, et al High-content, high-throughput screening for the identification of cytotoxic compounds based on cell morphology and cell proliferation markers. PLoS One. 2014 1;9(2):e88338 10.1371/journal.pone.0088338 24505478 10.1371/journal.pone.0088338 PMC3914966

[18] Donato MT, Tolosa L, Jiménez N, Castell J V, Gómez-Lechón MJ. High-content imaging technology for the evaluation of drug-induced steatosis using a multiparametric cell-based assay. J Biomol Screen. 2012 3;17(3):394–400. 10.1177/1087057111427586 22116976 10.1177/1087057111427586

[19] Emery A, Sorrell DA, Lawrence S, Easthope E, Stockdale M, Jones DO, et al A novel cell-based, high-content assay for phosphorylation of Lats2 by Aurora A. J Biomol Screen. 2011;16(8):925–31. 10.1177/1087057111413923 21788394 10.1177/1087057111413923

[20] Rao TD, Rosales N, Spriggs DR. Dual-Fluorescence Isogenic High-Content Screening for MUC16/CA125 Selective Agents. Mol Cancer Ther. 2011;10(10):1939–48. 10.1158/1535-7163.MCT-11-0228 21817115 10.1158/1535-7163.MCT-11-0228 PMC3191303

[21] Lecun Y, Bengio Y, Hinton G. Deep learning. Nature. 2015;521(7553):436–44. 10.1038/nature14539 26017442 10.1038/nature14539

[22] Angermueller C, Pärnamaa T, Parts L, Stegle O. Deep learning for computational biology. Mol Syst Biol. 2016;12(7):878 doi: 10.15252/msb.20156651 27474269 10.15252/msb.20156651 PMC4965871

[23] Esteva A, Kuprel B, Novoa RA, Ko J, Swetter SM, Blau HM, et al Dermatologist-level classification of skin cancer with deep neural networks. Nature. 2017;542(7639):115–8. 10.1038/nature21056 28117445 10.1038/nature21056 PMC8382232

[24] Pärnamaa T, Parts L. Accurate Classification of Protein Subcellular Localization from High-Throughput Microscopy Images Using Deep Learning. G3 (Bethesda). 2017;7(5):1385–92. 28391243 10.1534/g3.116.033654 PMC5427497

[25] Mamoshina P, Vieira A, Putin E, Zhavoronkov A. Applications of Deep Learning in Biomedicine. Mol Pharm. 2016;13(5):1445–54. 10.1021/acs.molpharmaceut.5b00982 27007977 10.1021/acs.molpharmaceut.5b00982

[26] Kandaswamy C, Silva LM, Alexandre LA, Santos JM. High-Content Analysis of Breast Cancer Using Single-Cell Deep Transfer Learning. J Biomol Screen. 2016;21(3):252–9. 10.1177/1087057115623451 26746583 10.1177/1087057115623451

[27] Kraus OZ, Grys BT, Ba J, Chong Y, Frey BJ, Boone C, et al Automated analysis of high-content microscopy data with deep learning. Mol Syst Biol. 2017;13(4):924 doi: 10.15252/msb.20177551 28420678 10.15252/msb.20177551 PMC5408780

[28] Eulenberg P, Köhler N, Blasi T, Filby A, Carpenter AE, Rees P, et al Reconstructing cell cycle and disease progression using deep learning. Nat Commun. 2017;8(1):1–6. 28878212 10.1038/s41467-017-00623-3 PMC5587733

[29] Ren S, He K, Girshick R, Sun J. Faster R-CNN: Towards real-time object detection with region proposal networks In: Advances in neural information processing systems. 2015 p. 91–9. 10.1109/TPAMI.2016.2577031 27295650

[30] Girshick R, Donahue J, Darrell T, Malik J. Region-Based Convolutional Networks for Accurate Object Detection and Segmentation. IEEE Trans Pattern Anal Mach Intell. 2016;38(1):142–58. 10.1109/TPAMI.2015.2437384 26656583 10.1109/TPAMI.2015.2437384

[31] D’Amato G, Luxán G, Monte-nieto G, Martínez-B, Torroja C, Walter W, et al Sequential Notch activation regulates ventricular chamber development. Nat Cell Biol. 2016;18(1):7–20. 10.1038/ncb3280 26641715 10.1038/ncb3280 PMC4816493

[32] Takemoto Y, Ramirez RJ, Yokokawa M, Kaur K, Ponce-Balbuena D, Sinno MC, et al Galectin-3 Regulates Atrial Fibrillation Remodeling and Predicts Catheter Ablation Outcomes. JACC Basic to Transl Sci. 2016;1(3):143–54. 10.1016/j.jacbts.2016.03.003 PMC4979747 27525318

[33] Simonyan K, Zisserman A. Very Deep Convolutional Networks for Large-Scale Image Recognition. arXiv Prepr [Internet]. 2014;arXiv:1409.1556. http://arxiv.org/abs/1409.1556

[34] LeCun Y, Bottou L, Bengio Y, Haffner P. Gradient-based learning applied to document recognition. In: Proceedings of the IEEE. IEEE; 1998. p. 2278–324.

[35] Zeiler MD. ADADELTA: An Adaptive Learning Rate Method. arXiv e-prints [Internet]. 2012;arXiv:1212.5701. http://arxiv.org/abs/1212.5701

[36] Long J, Shelhamer E, Darrell T. Fully convolutional networks for semantic segmentation. In: Proceedings of the IEEE conference on computer vision and pattern recognition. 2015. p. 3431–40. 10.1109/TPAMI.2016.2572683 27244717

[37] Pan SJ, Yang Q. A Survey on Transfer Learning. IEEE Trans Knowl Data Eng. 2010 10;22(10):1345–59.

[38] Zhang XD. Illustration of SSMD, z Score, SSMD*, z* Score, and t Statistic for Hit Selection in RNAi High-Throughput Screens. J Biomol Screen. 2011 8;16(7):775–85. 10.1177/1087057111405851 21515799 10.1177/1087057111405851

[39] Vysochanskij DF, Petunin YIJ. Justification of the 3-Sigma Rule for Unimodal Distribution. Theory Probab Math Stat. 1980;(21):25–36.

[40] Chung N, Zhang XD, Kreamer A, Locco L, Kuan PF, Bartz S, et al Median absolute deviation to improve hit selection for genome-scale RNAi screens. J Biomol Screen. 2008;13(2):149–58. 10.1177/1087057107312035 18216396 10.1177/1087057107312035

[41] Birmingham A, Selfors LM, Forster T, Wrobel D, Kennedy CJ, Shanks E, et al Statistical methods for analysis of high-throughput RNA interference screens. Nat Methods. 2009 8;6(8):569–75. 10.1038/nmeth.1351 19644458 10.1038/nmeth.1351 PMC2789971

[42] Held M, Schmitz MH a, Fischer B, Walter T, Neumann B, Olma MH, et al CellCognition: time-resolved phenotype annotation in high-throughput live cell imaging. Nat Methods. 2010 8;7(9):747–54. 10.1038/nmeth.1486 20693996 10.1038/nmeth.1486

[43] Mullarky E, Lucki NC, Beheshti R, Anglin JL, Gomes AP, Nicolay BN, et al Correction for Mullarky et al., Identification of a small molecule inhibitor of 3-phosphoglycerate dehydrogenase to target serine biosynthesis in cancers. Proc Natl Acad Sci. 2016;113(11):E1585–E1585. 10.1073/pnas.1602228113 26951666 10.1073/pnas.1602228113 PMC4801277

[44] Wilkinson L, Friendly M. The History of the Cluster Heat Map. Am Stat. 2008;63(2):179–184.

[45] Motulsky HJ, Christopoulos A. Fitting models to biological data using linear and nonlinear regression: A practical guide to curve fitting Fitting Model to Biol data using linear nonlinear Regres A Pract Guid to curve fitting. 2004;1–351.

[46] Chollet F, others. Keras [Internet]. GitHub; 2015. https://keras.io/

[47] The Theano Development Team, Al-Rfou R, Alain G, Almahairi A, Angermueller C, Bahdanau D, et al. Theano: A Python framework for fast computation of mathematical expressions. arXiv e-prints [Internet]. 2016 May;arXiv:1605.02688. http://arxiv.org/abs/1605.02688

[48] Jia Y, Shelhamer E, Donahue J, Karayev S, Long J, Girshick R, et al. Caffe: Convolutional architecture for fast feature embedding. In: Proceedings of the 22nd ACM international conference on Multimedia. 2014. p. 675–8.

