# Supplementary figures and images for: Tox_(R)CNN: Deep learning-based nuclei profiling tool for drug toxicity screening
Source: PLoS Comput Biol. 2018 Nov 30;14(11):e1006238. doi: 10.1371/journal.pcbi.1006238 (PMC6291153; doi:10.1371/journal.pcbi.1006238)

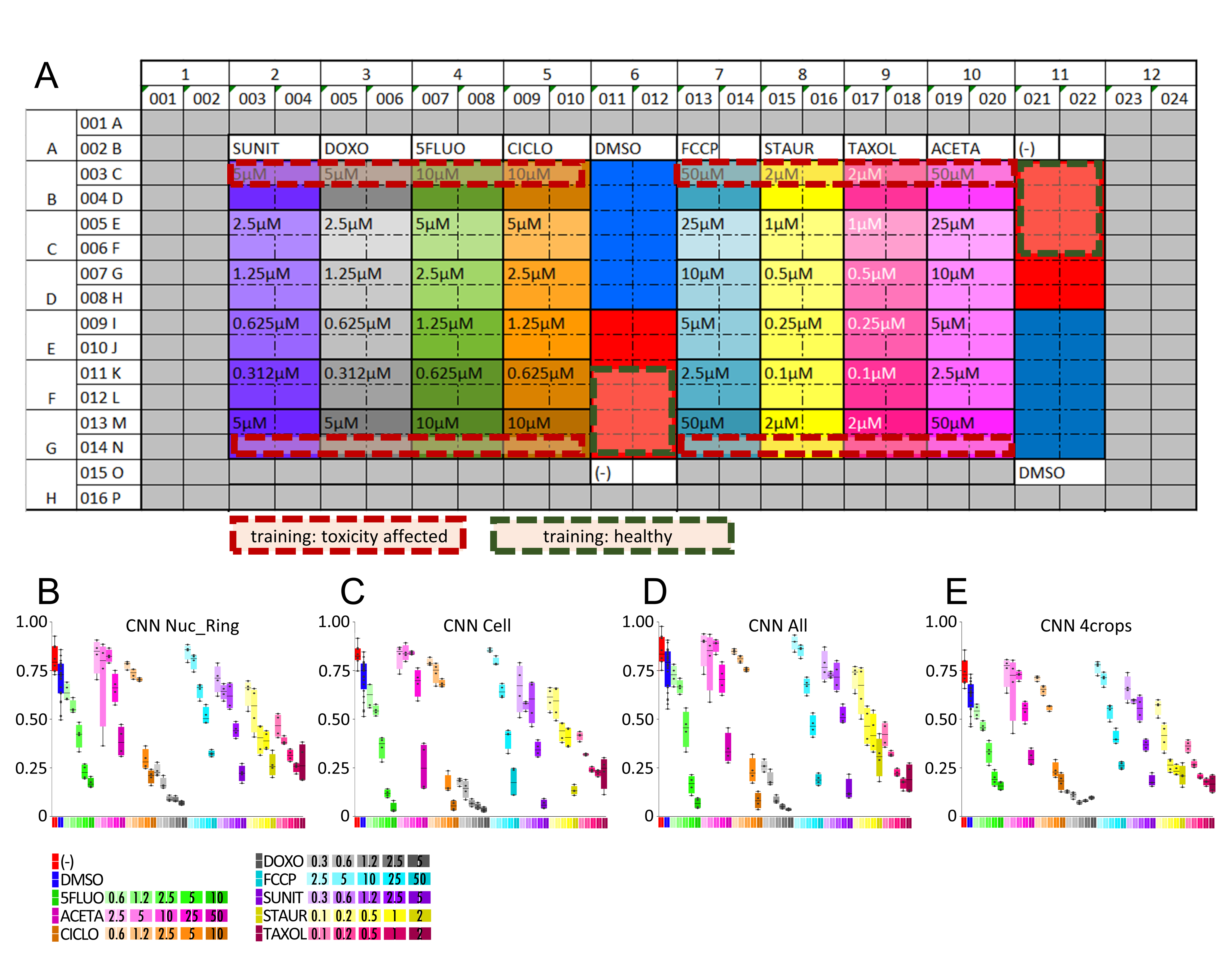

Supplement: S1 Fig — (A) Plate layout corresponding to a reference experiment used for both training and testing CNNs, where cells from indicated wells were used to create the training dataset with healthy (green) and toxicity affected (red) labelled cells coming from untreated wells and wells treated with the highest drug concentrations, respectively. (B-E) HL1 cells treated or not (-) with DMSO or the indicated concentrations of drugs (μM) from Experiment #1 were processed as described in the Materials and Methods. Boxplots of per-well toxicity assessments from CNN-based predictions: percentage of cells classified as healthy by the CNN Nuc_Ring (B), CNN Cell (C), CNN All (D), and CNN 4crops (E) models. (TIF) [file pcbi.1006238.s001.tif]

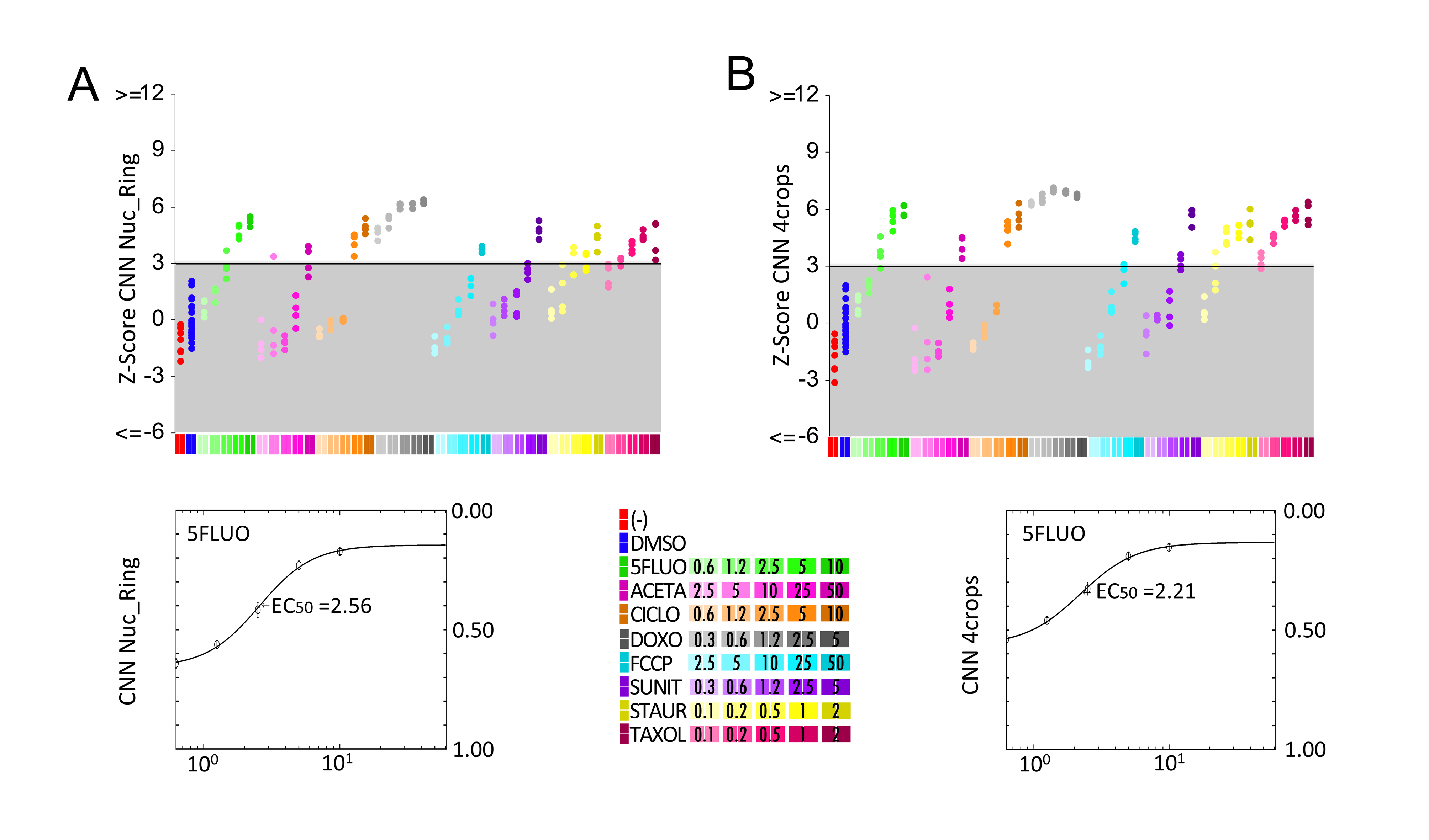

Supplement: S2 Fig — HL1 cells treated or not (-) with DMSO or the indicated concentrations of drugs (μM) from Experiment #1 were processed as described in the Materials and Methods. Plots display individual well toxicity readouts (top) and the 5-Fluorouracil dose-response curve (bottom), including the EC50, from CNN Nuc_Ring (A) and CNN 4crops (B) toxicity predictions. For each well, toxicity readouts were obtained by computing Z-scores (normalizing to DMSO-treated wells) with adjustment of the sign to display toxic effects as positive values. Z-scores > 3 represent toxic hits. (TIF) [file pcbi.1006238.s002.tif]

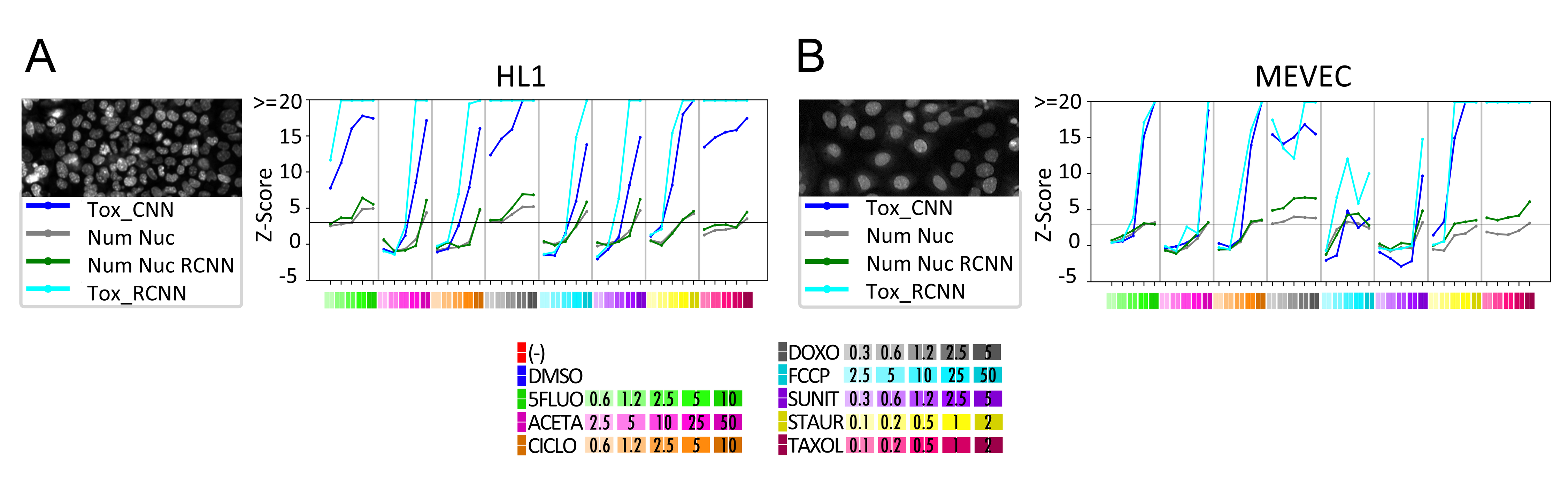

Supplement: S3 Fig — HL1 (A) and MEVEC (B) cells treated or not (-) with DMSO or the indicated concentrations of drugs (μM) were processed as described in the Materials and Methods (Experiments #2 and #10). Representative images are shown of untreated cells. Plots display mean toxicity readouts of four replicate wells, obtained from the percentage of healthy cells predicted by the CNN Nuc (Tox_CNN) or RCNN (Tox_RCNN) mixed models, and from nuclei counting by standard image segmentation (Num Nuc), or by RCNN-based automated detection (Num Nuc RCNN). For each well, toxicity readouts were obtained by computing Z-scores (normalizing to DMSO-treated wells) with adjustment of the sign to display toxic effects as positive values. (TIF) [file pcbi.1006238.s003.tif]

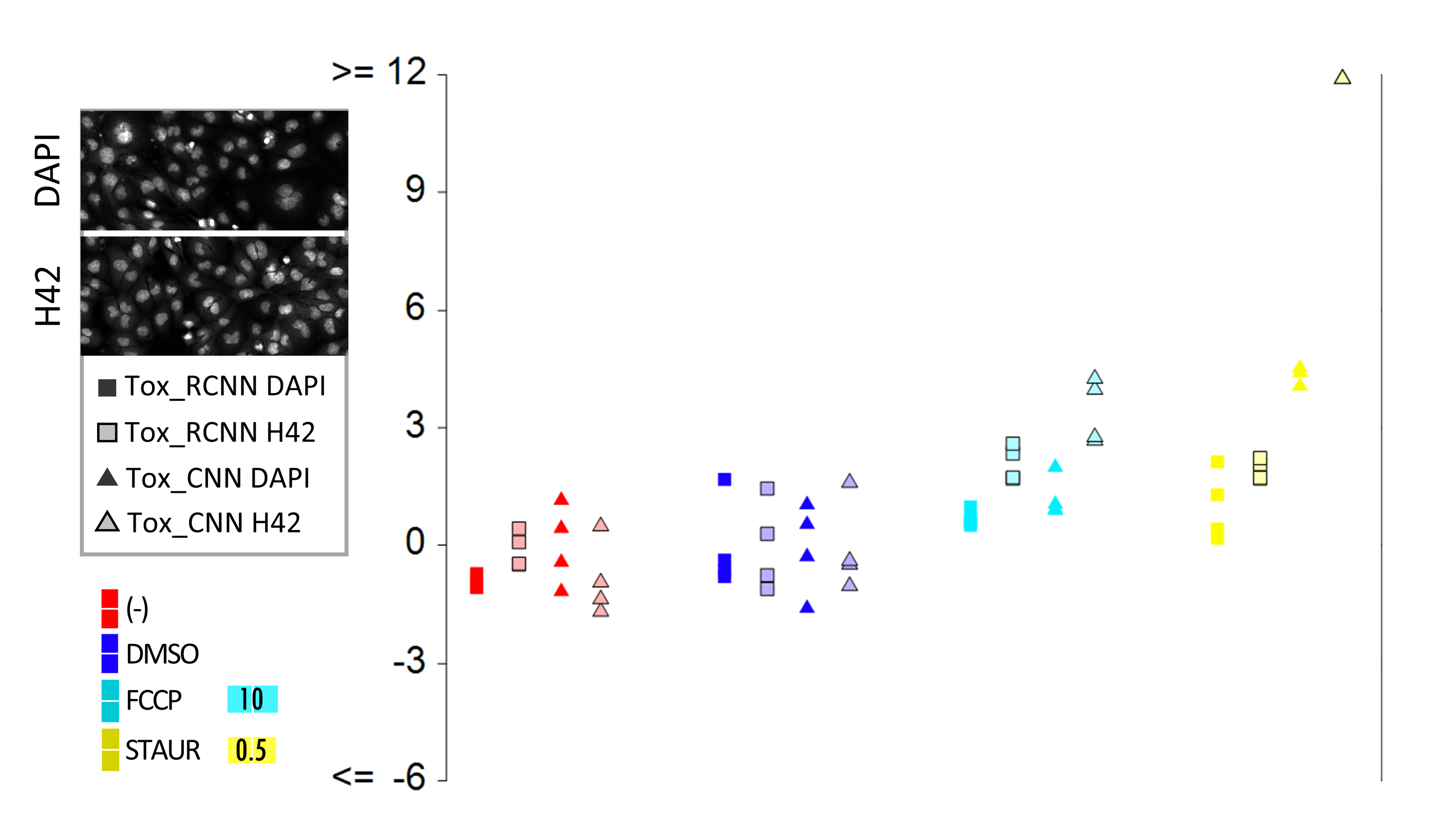

Supplement: S4 Fig — HL1 cells treated or not (-) with DMSO or the indicated concentrations of drugs (μM) were stained in parallel with DAPI (Experiment #26) or H42 (Experiment #27) as described in the Materials and Methods. Representative images of untreated cells are shown. Plots display toxicity readouts of four replicate wells, obtained from the percentage of healthy cells predicted by the CNN Nuc (Tox_CNN) or RCNN (Tox_RCNN) mixed models for both experiments. For each well, toxicity readouts were obtained by computing Z-scores (normalizing to DMSO-treated wells) with adjustment of the sign to display toxic effects as positive values. (TIF) [file pcbi.1006238.s004.tif]

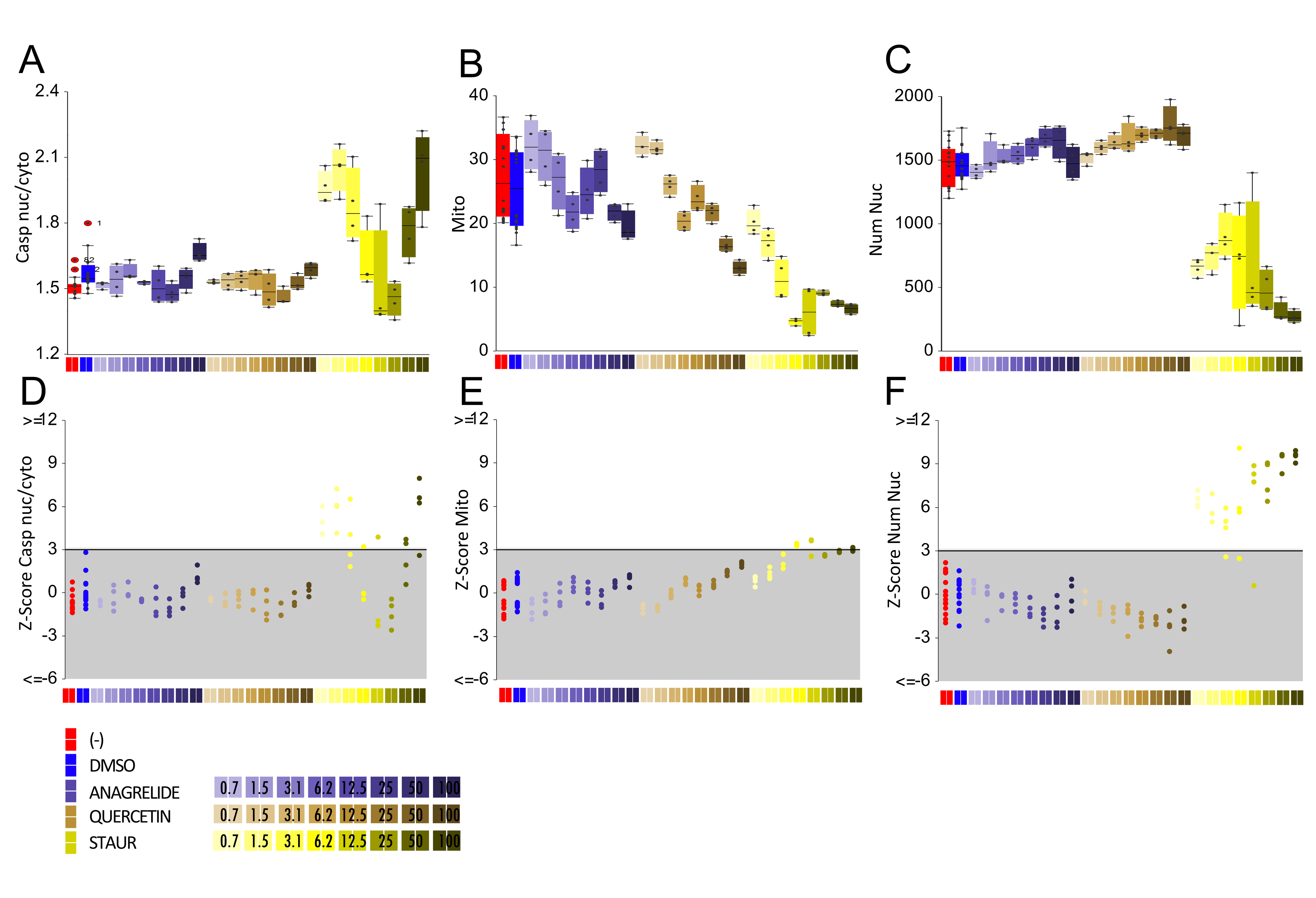

Supplement: S5 Fig — Primary cardiac fibroblasts (Experiment #25) treated or not (-) with DMSO or the indicated concentrations of drugs (μM) were processed as described in the Materials and Methods. Boxplots of per-well toxicity assessments in culture wells from established measurements (A-C), and corresponding individual well toxicity readouts (D-F), obtained from Caspase 3/7 nucleus:cytoplasm ratio (Casp Nuc/Cyto) (A,D), Mitotracker cytoplasmic intensity (Mito) (B,E), and nuclei counting (Num Nuc)(C,F). Data are from 4 replicate wells of the same experiment. For each well, toxicity readouts (D-F) were obtained by computing Z-scores (normalizing to DMSO-treated wells) with adjustment of the sign to display toxic effects as positive values. (TIF) [file pcbi.1006238.s005.tif]

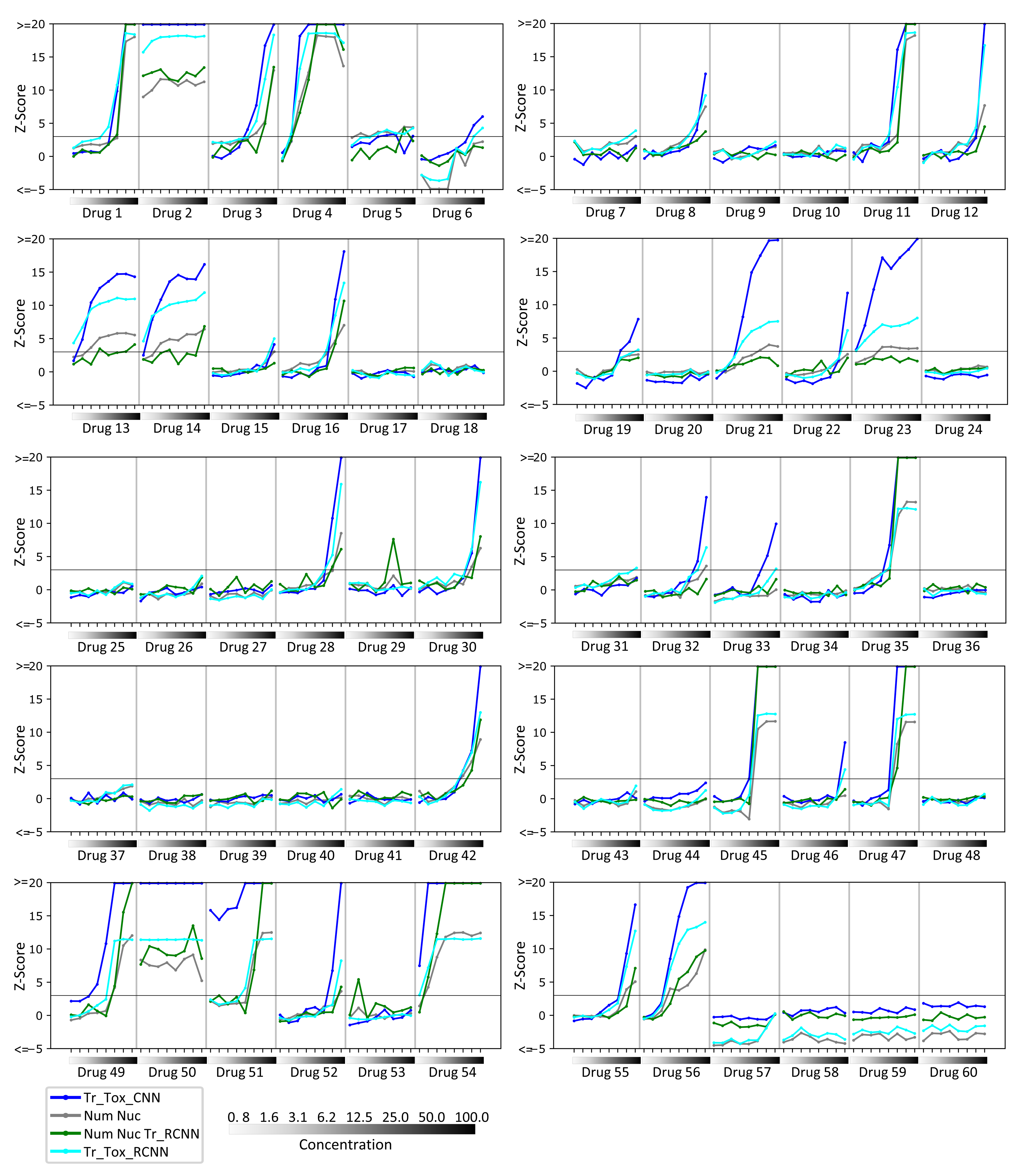

Supplement: S6 Fig — Pancreatic CAFs (Experiments #15–24) treated with 60 compounds at the indicated concentrations (μM) were processed as described in the Materials and Methods. Plots correspond to results in all 10 complete plates, displaying mean toxicity readouts of four replicate wells, obtained from the percentage of healthy cells predicted by the CNN (Tr_Tox_CNN) and RCNN (Tr_Tox_RCNN) mixed models after transfer learning, and from nuclei counting by standard image segmentation (Num Nuc), or by RCNN-based automated detection (Num Nuc Tr_RCNN). For each well, toxicity readouts were obtained by computing Z-scores (normalizing to DMSO-treated wells) with adjustment of the sign to display toxic effects as positive values. (TIF) [file pcbi.1006238.s006.tif]
